# Beyond the Metal Core: Platinum Incorporation Reshapes Redox Levels and Ligand Shells in Au**_25_** Thiolate Clusters

**DOI:** 10.1021/acsomega.6c07603

**Published:** 2026-09-17

**Authors:** Konstanty Zdunek, Tatiana Korona, Agnieszka Więckowska

**Affiliations:** Faculty of Chemistry, 49605 University of Warsaw, Pasteura 1, Warsaw 02-093, Poland

## Abstract

Platinum incorporation into atomically precise Au_25_ thiolate clusters is known to strongly affect their electronic structure, but experimental studies of these systems are often complicated by the simultaneous formation of Au_25_ and Au_24_Pt species. Here, we examine butanethiolate- and hexanethiolate-protected Au_25_/Au_24_Pt cluster mixtures obtained by a Brust–Schiffrin-type synthesis, without applying oxidative purification conditions that caused substantial degradation of the platinum-containing products. The overall Au/Pt composition of the products was quantified by ICP-MS, while TEM and DLS were used to characterize their particle-size distributions. The Au/Pt ratios indicate that platinum incorporation competes with the formation of larger platinum-rich nanoparticles and reveal differences in product distribution between the butanethiolate- and hexanethiolate-protected systems. UV-Vis and square-wave voltammetry measurements reveal reproducible differences between the platinum-containing mixtures and the corresponding Au_25_ reference systems, while FT-IR spectroscopy shows ligand-dependent changes in the vibrational signatures of the alkylthiolate shell. DFT calculations reproduce the stronger core distortion expected for Au_24_Pt relative to Au_25_ and provide a qualitative framework for interpreting the experimental trends. Rather than assigning intrinsic properties to isolated Au_24_Pt clusters, the present work shows how platinum incorporation manifests itself in experimentally accessible Au_25_/Au_24_Pt mixtures. The results further show that these effects differ between the butanethiolate- and hexanethiolate-protected systems.

## Graphical abstract

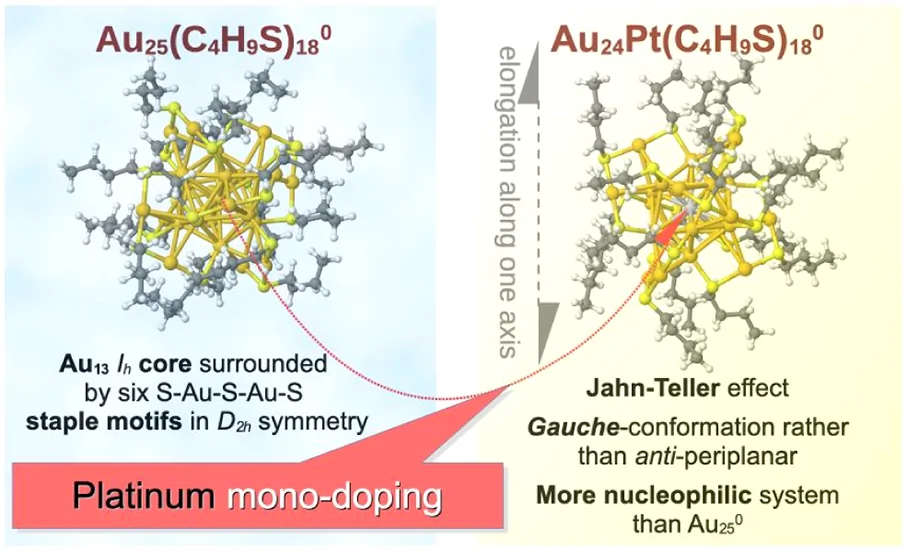

## Introduction

Gold clusters protected by thiolates exhibit a number of unique spectroscopic and electrochemical properties. In particular, they show remarkable catalytic activity in numerous reactions, including the electrooxidation and electroreduction of hydrogen peroxide,^1^ as well as in more conventional homogeneous catalytic processes.^2^ Their incorporation into selected architectures can modify the mechanisms of biocatalytic reactions, for example, in fructose oxidation.^3^ Because of the high hydrophobicity of alkanethiolate ligands, such clusters can also be embedded into lipid monolayers,^4^ which makes them useful as models of cell membranes.^5^ Despite the presence of strongly bound thiolates, thiolate-protected gold clusters have also been proposed as drug carriers.^6^ As a result, gold-containing clusters are attractive in many areas of electrochemistry and biochemistry.

Structures of the prototypical Au_25_(RS)_18_ (Au_25_) and its monoplatinum-doped analogue Au_24_Pt­(RS)_18_ (Au_24_Pt) have been described in detail by X-ray diffraction (for Au_25_)^7^ and by mass spectrometry,^8^ NMR^2^, and EXAFS^9^ (for Au_24_Pt vs Au_25_).^10^ The Au_25_ cluster in particular has been extensively investigated with respect to its synthesis,^2,11–13^ formation mechanism,^14^ and spectral^15^ and electrochemical properties.^16^ The Au_24_Pt cluster, first obtained in 2012,^2,9^ has also been the subject of several studies, but some reported features remain controversial. For example, the shapes of UV-Vis spectra depend sensitively on the purification protocol used for Au_24_Pt samples.^2,17^

The Au_25_ and Au_24_Pt clusters adopt closely related core-shell structures. Both may be viewed as ultrasmall metal nanoparticles with an icosahedral Au_12_M core (M = Au or Pt), surrounded by six RS-Au-RS-Au-RS staple motifs. Figure 1 shows the numbering convention of the metal atoms in Au_24_M clusters, adapted from a proposition for a convention for numbering vertices of the icosahedron.^18^ The core comprises 12 surface gold atoms (1–12) and a central atom (13, either Au or Pt) trapped inside the icosahedral cage formed by the surface atoms.^5,9^ If the core geometry were perfectly regular, the metal framework would exhibit ideal *I*_*h*_ symmetry.^7^ Each of the 12 surface gold atoms also binds to the terminal sulfur atoms from the staple motifs, giving rise to a second class of gold atoms, sometimes called *staple* or *stellated* atoms.^5,9^

**Figure 1:**
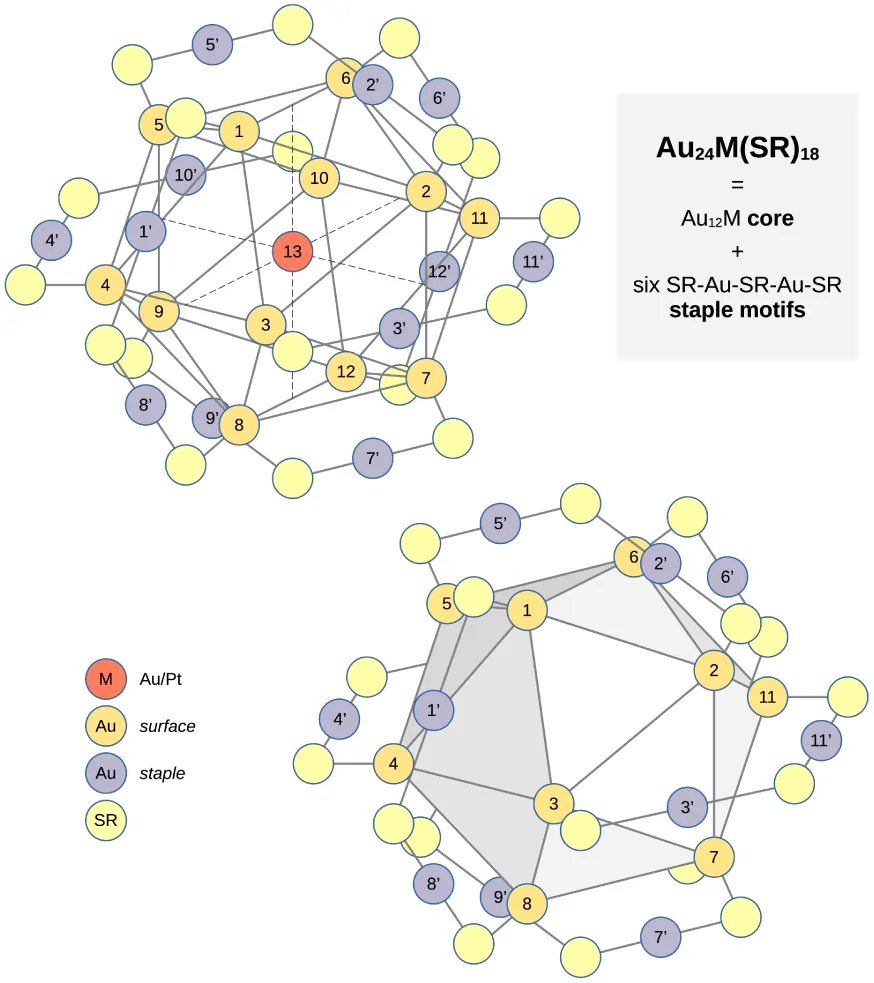
Numbering convention of gold and platinum atoms in Au_24_M­(RS)_18_ clusters (M = Au or Pt).

Symmetry considerations indicate that if surface-atom pairs related as the 1,8 pair are connected by the same staple motif, the corresponding staples adopt orientations lying approximately in three mutually orthogonal planes (Figure 1). If the staple motifs were completely planar and attached to an ideal *I*_*h*_ icosahedron (disregarding conformational degrees of freedom of the hydrocarbon chains), the overall structure would display *D*_*2h*_ symmetry, i.e., a subgroup of the octahedral *O*_*h*_ symmetry, with eight triangular core faces remaining exposed.^7^ In general, Au_24_M clusters are anisotropic objects, in contrast to conventional, nearly spherical metal nanoparticles. Their anisotropy and ultrasmall size are directly linked to their high catalytic and electrocatalytic activities.^19^

Within the Au_12_M core, the 12 surface gold atoms interact both with the central atom M and with each other. These Au–Au contacts are often interpreted in terms of directional, partially covalent interactions associated with 5d-6s hybridization.^20,21^ In Au_24_Pt, the Au–Pt contacts are even stronger than the aurophilic Au–Au interactions, partly because of the finite electronegativity difference between gold and platinum. This leads to bond contraction and partial loss of 5d-6s hybridization, leaving a larger fraction of 5d electron density available for interaction with the environment.^9^

Owing to electron delocalization within the core, Au–Au and Au–Pt bonds are better described as components of a delocalized network.^20^ This justifies the application of the superatom concept, derived from the *jellium* model,^22,23^ in which a subset of valence electrons is confined in a quasi-spherical potential well defined by formally cationic Au^+^ centers. In Au_13_ and Au_12_Pt cores extracted from neutral Au_25_^0^ and Au_24_Pt^0^ clusters, there are formally six Au^+^ atoms arising from bonding to thiolate ligands. Each of the 18 RS^–^ groups requires bonding to one Au^+^ center, which accounts for all 12 staple atoms and six of the surface atoms. The remaining seven (13–6) neutral gold atoms in Au_25_^0^ formally contribute one 6s electron each, yielding seven delocalized electrons. These occupy superatomic 1S and 1P orbitals of s- and p-like symmetry. In Au_24_Pt^0^, the Pt center is generally assumed not to contribute to this delocalized electron count,^24,25^ so the Au_12_Pt core contains six delocalized electrons.

When the superatomic 1S^2^1P^6^ configuration is realized, as in Au_25_^–^ or Au_24_Pt^2–^, the resulting octet of delocalized electrons leads to particularly stable, closed-shell systems for which the three 1P orbitals can remain nearly degenerate. For differently charged species, including neutral Au_25_^0^ and Au_24_Pt^0^, the partially filled 1P manifold undergoes Jahn–Teller splitting into a lower-energy doubly degenerate level and a higher-energy non-degenerate level (Figure S9). The Jahn–Teller distortion is reflected, among other ways, in deviations of the core geometry from ideal *I*_*h*_ symmetry.^17,21^ Similar symmetry-lowering deformations may occur in octahedral coordination structures under specific conditions. At a highly simplified level, some analogies may be drawn between distortions of molecules exhibiting *I*_*h*_ and *O*_*h*_ symmetries, as *I*_*h*_ can be considered as an evolved form of *O*_*h*_; however, *I*_*h*_ is not the parent group of *O*_*h*_ (Figure S10). In contrast to standard coordination compounds, Au_25_ and Au_24_Pt clusters are capable of participating in multiple electron transfers, resulting in distinct superatomic configurations. The ability of clusters to accept and donate additional delocalized electrons is directly observable as multiple electrooxidation and electroreduction waves,^11^ and Au_25_^–^ and Au_24_Pt^0^ are commonly taken as reference species for discussing their redox series, i.e., oxidations (O1, O2, etc.) and reductions (R1, R2, etc.).^22,23^

The standard synthesis of Au_25_^0^ and Au_24_Pt^0^ clusters involves reduction of the corresponding metal precursors (HAuCl_4_ for Au_25_, HAuCl_4_/H_2_PtCl_6_ for Au_24_Pt) by NaBH_4_ in a one-phase THF/TOABr system.^8,13,17,26^ The initial stages of the reaction comprise nucleation, i.e., rapid core formation upon addition of NaBH_4_,^14^ followed by limited core growth, which is curtailed by the presence of a large excess of thiol (thiol:Au ≈ 6:1 typically)^26^ and passivation via formation of staple motifs (during HAuCl_4_ + RSH interaction).^14^ Conducting the reaction in a single organic phase accelerates cluster formation relative to the original two-phase Brust–Schiffrin protocol.^13,26^ The crude product contains a mixture of clusters and nanoparticles and requires extensive purification, including removal of polar impurities by water-toluene extraction,^17^ elimination of excess thiols (centrifugation in light alcohols),^2^ and, crucially, oxidation of Au_25_^–^ to Au_25_^0^ while preserving the neutral Au_24_Pt^0^ species formed during the reaction.^2,5^ Larger nanoparticles need to be separated from clusters of the desired ultrasmall size.^27–29^ Separation of Au_24_Pt^0^ from Au_25_^0^ has been attempted by specialized chromatographic methods and by treatment with H_2_O_2_, exploiting the higher thermodynamic stability of neutral Au_24_Pt^0^ relative to Au_25_^0^ predicted from superatomic arguments.^2,8,25^

In practice, isolation of Au_24_Pt from Au_25_-containing products is challenging, and under the conditions used in this study, oxidative purification caused substantial degradation of the platinum-containing material. We therefore examined cluster-enriched Pt-containing products obtained without oxidative purification. Although such mixtures do not permit fully species-specific characterization or determination of the intrinsic properties of isolated Au_24_Pt clusters, they provide experimental access to Pt-containing cluster products without applying purification procedures that alter or degrade the platinum-containing fractions.

Comparison of these products with ligand-matched Au_25_ reference samples allows for reproducible spectroscopic and electrochemical differences to be identified under experimentally accessible conditions. By combining ICP-MS analysis of the overall Au/Pt composition, particle-size characterization, UV-Vis and FT-IR spectroscopy, electrochemical measurements, and DFT calculations for model Au_25_ and Au_24_Pt clusters, we distinguish direct experimental observations for the mixtures from assignments that are conditional on the proposed sample composition. The scope and limitations of this approach are explicitly considered throughout the discussion. The ability to detect Pt-related spectroscopic and electrochemical effects without applying additional oxidative purification may also be practically relevant in applications where the preservation of the cluster-containing fraction is more important than complete separation of individual cluster species.

## Results and Discussion

### Composition of Au_25_ Reference Samples and Pt-Containing Products

Four syntheses of clusters protected by butanethiolate BuSH and hexanethiolate HxSH were performed: Au_25_^0^-Bu, Au_25_^0^-Hx, Au_24_Pt^0^-Hx (in the mixture), and Au_24_Pt^0^-Bu (in the mixture). Platinum-containing products were divided into fractions with decreasing amounts of the smallest clusters based on differences in solubility in acetonitrile.^27–29^ The purest, first fractions containing mainly Au_24_Pt, Au_25_, and platinum nanoparticles were analyzed for their composition by ICP-MS, measuring the total gold and platinum content. Direct mass-spectrometric quantification of the Au_25_:Au_24_Pt ratio is problematic because the ionization efficiencies of the two species are not necessarily identical (the ratio of the Au_25_ and Au_24_Pt signal intensities does not have to correspond with the molar ratio).^29^ ESI-QTOF-MS analysis of the BuSH-protected Pt-containing sample revealed a molecular-ion envelope consistent with Au_25_-Bu, whereas no unambiguous molecular-ion signal attributable to Au_24_Pt-Bu was detected under the applied conditions (Figure S2). Consequently, ESI-MS was not used as direct evidence for the presence or abundance of the proposed Au_24_Pt component. Table 1 presents the Ξ (molar ratio Au:Pt) values resulting from the applied molar ratio of HAuCl_4_ and H_2_PtCl_6_ for substrates and the values recorded for products by ICP-MS.

**Table 1:** The Ξ Values (Molar Ratio Au:Pt) for Obtained Products Containing Au_24_Pt Clusters

	Ξ for substrates	Ξ for products
Au_24_Pt-Hx + Au_25_-Hx and PtNPs	3.9:1	8.97:1
Au_24_Pt-Bu + Au_25_-Bu and PtNPs	3.7:1	4.84:1

The ICP-MS results demonstrate that the investigated materials are multicomponent systems containing Au_25_ clusters, Pt-containing clusters, including Au_24_Pt, and platinum-rich nanoparticles. Accordingly, all subsequent spectroscopic and electrochemical measurements should be interpreted as responses of Au_25_/Au_24_Pt/PtNP mixtures rather than isolated Au_24_Pt clusters. The multicomponent nature of the samples necessarily limits species-specific assignments. ICP-MS provides the overall Au/Pt ratio but does not independently determine the relative abundance of individual cluster species, while TEM and DLS provide information primarily on particle size rather than molecular composition. Therefore, assignments of spectroscopic and electrochemical features to the Au_24_Pt component are based on comparison with the corresponding Au_25_ reference samples, literature data, and DFT calculations and should be interpreted within these limitations. The strategy used to obtain a product containing both Au_25_ and Au_24_Pt clusters, without separating them, leads to an increased proportion of gold atoms relative to platinum in the final mixture. This indicates that a significant fraction of platinum atoms is lost as larger nanoparticles, which can be separated on a chromatographic column packed with 60 Å silica gel and have sizes exceeding 6 nm. The higher Ξ value observed for the Au_24_Pt-Hx sample relative to Au_24_Pt-Bu indicates a different distribution of platinum among the products formed during synthesis. Together with the ICP-MS results, TEM and DLS measurements indicate the presence of platinum-containing nanoparticles in addition to atomically precise clusters. These particles, with sizes below 6 nm, are present in the studied samples. According to DLS and TEM results, the first fraction after acetonitrile extraction, examined by ICP-MS, contains the smallest clusters with sizes of approximately 1–2 nm (Figure 2), which is consistent with optimized geometries of Au_25_ and Au_24_Pt from DFT calculations.

**Figure 2:**
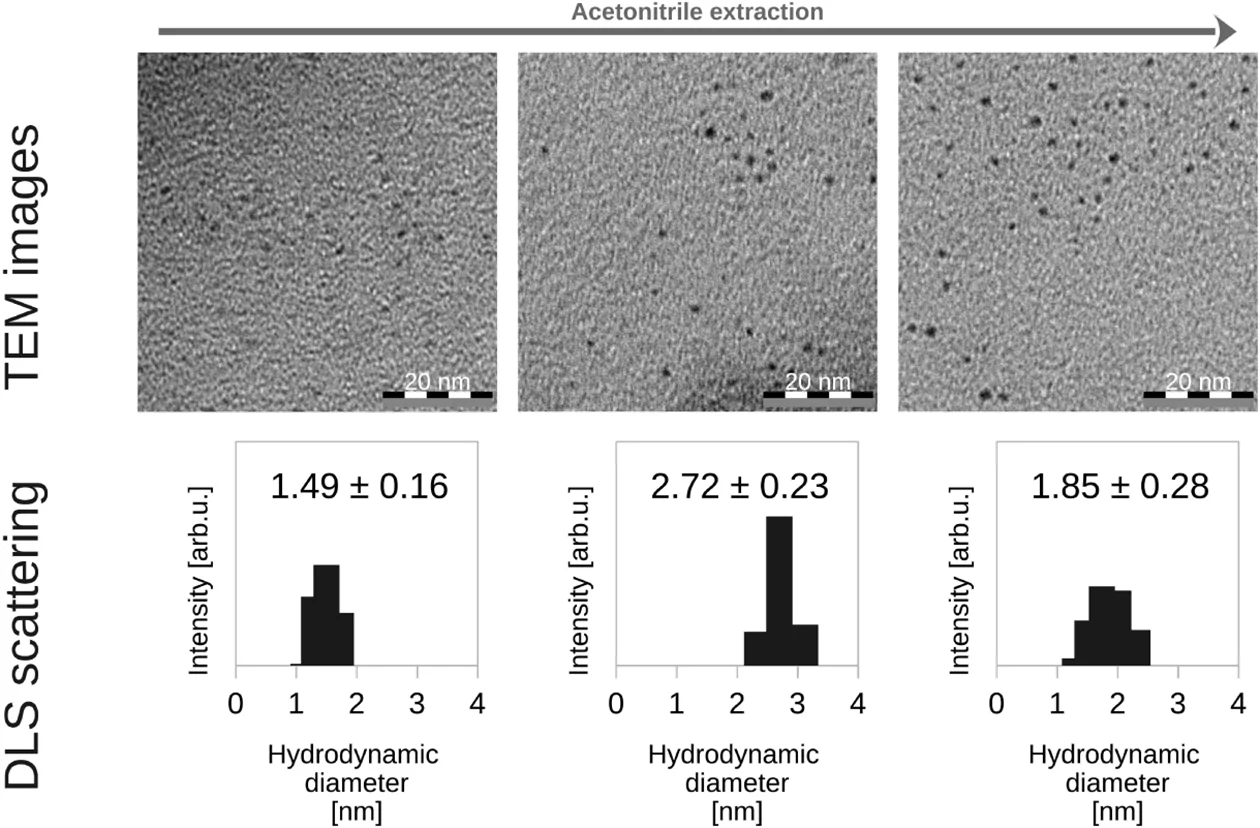
TEM images (bright-field imaging in solid phase) and DLS results (0.005 mg/mL toluene solutions, 1 cm optical cell) with average hydrodynamic diameters for the first three fractions of Au_24_Pt-Hx-containing samples (Hx, hexane-1-thiolate).

The calculated Au_25_:Au_24_Pt:Pt_k_ ratios indicate that the ligand structure significantly affects product distribution during synthesis. To obtain an approximate compositional estimate, we considered a simplified model in which Au_25_, Au_24_Pt, and Pt-rich nanoparticles Pt_k_ are treated as the major metal-containing components. Under this assumption, and using the known reaction yields and calculations described in the Supporting Information, approximate Au_25_:Au_24_Pt:Ptk molar ratios of 3.9:1:8.5 for HxSH and 4.9:1:29.9 for BuSH are obtained. These values should be regarded as model-based estimates rather than as a complete quantitative description of all species present in the samples. Minor contributions from other gold-containing clusters, Au-containing or bimetallic nanoparticles, and metal-thiolate species cannot be excluded by the present characterization.

Under the applied synthesis conditions, the relative abundance of Au_24_Pt-containing products is higher for HxSH than that for BuSH. On the other hand, the nucleation–growth-passivation mechanism is mainly under kinetic control for pure gold clusters.^14^

Au_24_Pt^0^ is postulated to be thermodynamically more stable than Au_25_^0^.^25^ Within the two ligand systems examined here, the higher relative abundance of Au_24_Pt-containing products obtained with HxSH may reflect differences in the kinetics of nucleation, growth, and passivation. One possible contribution is the different mobility and packing behavior of BuSH- and HxSH-derived thiolates, which may affect the relative probabilities of Au–Au, Pt–Pt, and Au–Pt associations during cluster formation. Because only two alkanethiolates were investigated, however, the present results do not establish a general relationship between the alkyl-chain length and product distribution.

### Spectral Properties of Au_25_ and Au_24_Pt Clusters

The Au_25_^0^ cluster exhibits the characteristic UV-visible spectrum that differs from the Au_25_^–^ form, formed directly by reduction of the precursor (Figure 3). Au_25_^0^ exhibits characteristic signals (in hexane) at 396 nm (Au_25_-Bu) or 393 nm (Au_25_-Hx) and 688 nm (Au_25_-Bu) or 681 nm (Au_25_-Hx),^5,15,30^ indicating subtle ligand-dependent differences in the electronic structure of the clusters. Gaps between energy levels involved in electron transitions observed by UV-Vis spectra for Au_25_^0^-Hx are slightly greater than for Au_25_^0^-Bu.

**Figure 3:**
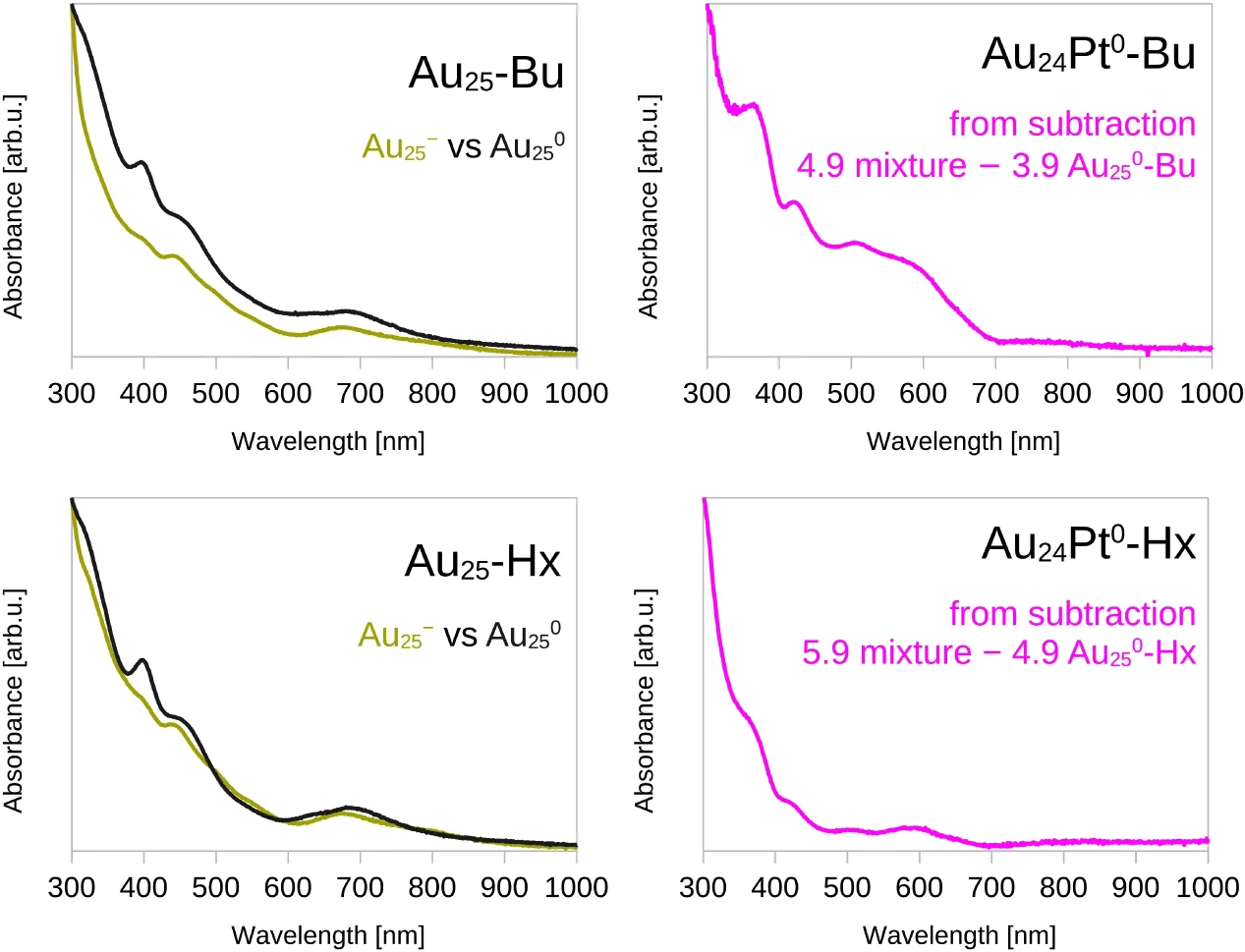
Left side: UV-Vis spectra (normalized, in hexane) of Au_25_^–^ vs Au_25_^0^ protected by butane-1-thiolate (Bu) and hexane-1-thiolate (Hx); right side: estimated spectral contributions of Au_24_Pt^0^-Bu and Au_24_Pt^0^-Hx obtained by subtraction from the corresponding Au25^0^/Au24Pt^0^ mixtures.

An estimated spectral contribution of the Au_24_Pt component was obtained by subtracting a suitably scaled spectrum of pure Au_25_ from the spectrum of the corresponding Au_25_/Au_24_Pt mixture, using the experimentally estimated Au_24_Pt:Au_25_ molar ratio (Figure 3). Averaged spectra of Au_24_Pt^0^, independent of the type of alkanethiolate, exhibit characteristic reproducible signals, approximately at 500 nm. The 580 nm band, obtained computationally in Au_24_Pt^0^-Hx, is postulated as an Au_24_Pt signature in several publications.^2,17^ The type of alkanethiolate does not affect the shapes of UV-Vis spectra of Au_25_^0^ significantly.^30^ Discrepancies - among the reported UV-Vis spectra for Au_24_Pt^0^ clusters result from different alkanethiolates, purification methods, impurities formed during synthesis, and, in this case, computational assumptions. Despite the limitations associated with spectral subtraction and estimated sample composition, the obtained spectral features are consistent with literature reports for Au_24_Pt clusters.^2,17^

In the *all*-*trans* conformation of the four-carbon (Bu) chain, the C_1_ and C_4_ atoms adopt a maximally extended arrangement, corresponding to an *anti* orientation in the Newman projection along the C_2_–C_3_ bond (dihedral angle of 180°). In contrast, the *gauche* conformation brings C_1_ and C_4_ into closer proximity, with the respective substituents adopting a *gauche* relationship characterized by a dihedral angle of 60°. For short thiols, both the *all*-*trans* and *gauche* conformations are typically considered low-energy arrangements of comparable stability.^31^ FT-IR results suggest a higher contribution of *gauche* conformations in Au_24_Pt^0^-Bu relative to Au_25_^0^-Bu. It was inferred from studying shifts of selected vibrational bands of methylene groups toward higher wavenumbers. This effect was observed only for the Au_24_Pt^0^-Bu sample (in the mixture) in comparison to Au_25_^0^-Bu, consistent with a higher contribution of *gauche*-like conformations (Figure S4). The largest shifts were obtained experimentally for NIR-range signals (second overtone region), with one of the highest peaks at 8380 cm^–1^ for Au_25_^0^-Bu and 8385 cm^–1^ for Au_24_Pt^0^-Bu. For hexanethiolate-protected clusters, no signal shifts between Au_25_^0^ and Au_24_Pt^0^ were observed. To further assess this interpretation, DFT calculations of the frequencies of some vibrations were performed (Figure 4).

**Figure 4:**
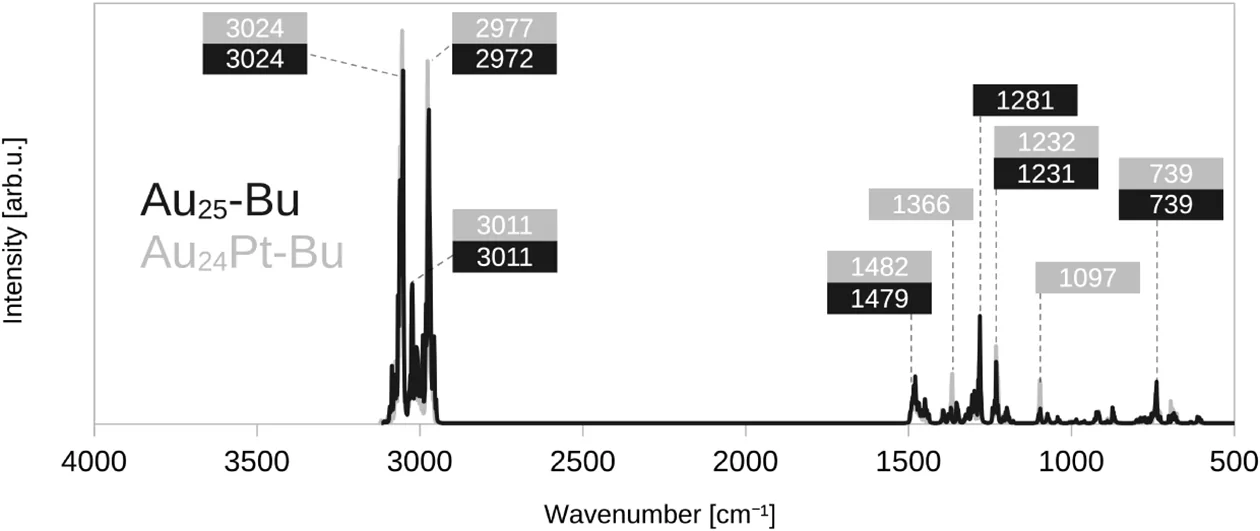
Wavenumbers of some vibrations, calculated with DFT, found in Au_25_^0^ and Au_24_Pt^0^ clusters protected by butane-1-thiolate (Bu), in vacuum.

### Electrochemical Properties of Au_25_ and Au_24_Pt Clusters

SWV data reveal a slight increase in the potential separation between adjacent peaks for the hexanethiolate-protected species compared with the butanethiolate-protected species. Similar to the UV-Vis data, the electrochemical results indicate subtle ligand-dependent differences in the electronic structure of the clusters, reflected by slightly larger separations between adjacent redox transitions for hexanethiolate-protected species.

The observed SWV signals coincide with those postulated in the literature. Characteristic O1–O2 oxidation doublets were observed both for Au_25_ and Au_24_Pt.^22,23^ The anodic shift of the O1 signal assigned to the Au_24_Pt component relative to Au_25_ indicates that oxidation of the Pt-containing cluster occurs at a higher potential, consistent with the modified electronic structure introduced by platinum incorporation. The difference between these signals is about 0.35 V (Table 2). The relative positions of the oxidation signals are consistent with literature reports indicating greater thermodynamic stabilization of Pt-doped clusters; however, in the present mixed samples the potential difference is small (ca. 0.02 V) and should be interpreted cautiously. Collectively, the platinum-containing system, having one fewer electron, is more difficult to oxidize, which manifests as an anodic shift of the observed potentials. Collectively, the electrochemical data indicate that platinum incorporation alters the redox properties of the cluster and shifts the oxidation processes toward more positive potentials.

**Table 2:** Potentials of SWV Signals O4–O1 and R1 (Successfully Recorded) of Au_25_ and Au_24_Pt Clusters Protected by Butane-1-Thiolate (Bu) and Hexane-1-Thiolate (Hx) (0.5 mg/mL + 0.1 M NBu_4_PF_6_ Dichloromethane Solutions, 25 Hz)

	O4	O3	O2	O1	R1
Au_25_-Bu	1.47 V	1.24 V	0.45 V	0.12 V	–1.50 V
Au_25_-Hx		1.26 V	0.44 V	0.11 V	–1.55 V
Au_24_Pt-Bu			0.79 V	0.46 V	–0.27 V
Au_24_Pt-Hx			0.79 V	0.47 V	–0.28 V

CV curves of the obtained products were also recorded (Figure 5, Figure S5). It can be seen that the analyzed oxidation-reduction processes of Au_25_ are Nernstian and reversible if the species between which the reaction occurs are stable.^16,17^ For example, the O1–O2 oxidation doublet exhibits a typical Nernstian behavior, and the peak heights are consistent with the Randles–Š]­evčík equation (Figure S5). Therefore, it can be seen that the Au_25_^–^, Au_25_^0^, and Au_25_^+^ species are reasonably stable in dichloromethane solutions. The Au_25_^3+^ state appears substantially less stable under the applied conditions, as indicated by the absence of a reversible electrochemical response. The CV curves of Au_24_Pt-containing samples show weak signals from Au_24_Pt, clearly visible in the SWV voltammograms (Figure 5).

**Figure 5:**
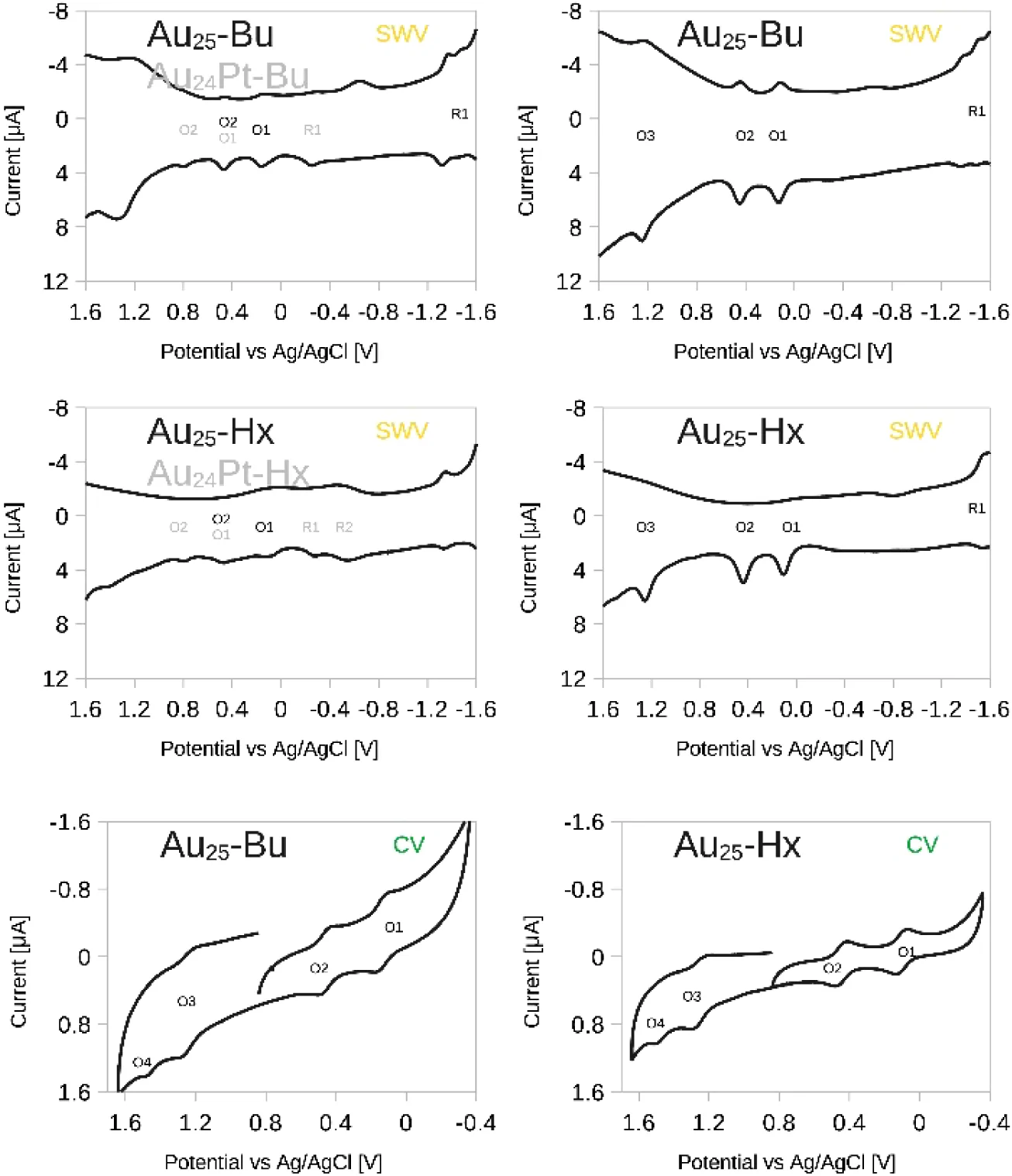
SWV voltammograms of the Pt-containing BuSH- and HxSH-protected samples (left) and the corresponding pristine Au_25_-Bu and Au_25_-Hx reference samples (right), together with CV voltammograms of pristine Au_25_-Bu and Au_25_-Hx (bottom). Black peak labels in the SWV traces indicate transitions assigned to the Au_25_ component, whereas gray labels indicate additional transitions assigned to the proposed Au_24_Pt component. Measurements were performed in dichloromethane containing 0.1 M NBu_4_PF_6_ (SWV: 25 Hz; CV: 20 mV s^–1^).

### DFT Calculations

The calculations for geometry optimization (Table 3) and vibrational frequencies (Figure 4) of Au_25_^0^-Bu and Au_24_Pt^0^-Bu species were performed with DFT using the B97 functional with the D3 dispersion correction.^32^ The starting geometries were taken either from crystallographic data (for Au_25_^0^-Bu)^8^ or by a modification of the DFT-optimized structure of Au_24_Pt­(CH_3_S)_18_ (for the Au_24_Pt^0^-Bu species).^2^ The TD-DFT calculations for UV-Vis spectra simulations (Figure S6) were performed with the CAM-B3LYP functional.^33^ A local minimum on the potential-energy surface was obtained for each optimized starting structure, as confirmed by the absence of imaginary harmonic frequencies. No exhaustive conformational search was performed, and the optimized geometries should therefore be regarded as representative local minima rather than as global-minimum ligand-shell conformations. Solvent, counterion, and thermal effects were not included explicitly in the calculations. For hydrogen, carbon, and sulfur atoms, the 6-31G­(d,p) orbital basis was applied,^34,35^ while for gold and platinum, the relativistic LanL2DZ basis was used.^36–38^ Due to the paramagnetism of the Au_25_^0^ cluster, the unrestricted formalism has been utilized, resulting in different α and β orbitals, while the restricted formalism has been applied for the closed-shell Au_24_Pt^0^ (Figure S7). The superatomic orbital assignments shown in Figure S7 follow the established orbital scheme for Au25-type clusters and are used here for qualitative interpretation; their energetic positions relative to the HOMO should not be regarded as universally fixed. Because the cluster-core symmetry is lowered relative to ideal *I_h_* symmetry, the calculated superatomic orbitals are distorted and contain mixed atomic-orbital contributions. In Au24Pt-Bu, HOMO–2 is the first among the higher-energy occupied orbitals showing nonzero contributions from s-type atomic functions centered on Pt and Au atoms, which further supports its assignment as the S-type superatomic orbital shown in Figure S7.

**Table 3:** Distances between Opposite Surface Gold Atoms in Au_13_ and Au_12_Pt (From Au_25_^0^ and Au_24_Pt^0^), Calculated as a Sum of *n*-M and *m*-M Distances (M = Au or Pt) for Each of the Six *n-m* Segments, from DFT Calculations

	1–12	2–9	3–10	4–11	5–7	6–8
Au_13_	6.01 Å	5.97 Å	5.78 Å	5.80 Å	5.97 Å	5.96 Å
Au_12_Pt	6.15 Å	5.77 Å	5.68 Å	5.90 Å	5.85 Å	5.86 Å

All calculations were performed with the *Gaussian* 16 program.^39^ To assess the energetic significance of the ligand-shell conformation, an additional all-anti Au_25_-Bu structure was optimized and compared with the Au_25_-Bu structure containing a partial contribution of gauche-like conformations. The differences in internal energy and enthalpy between the two structures were approximately 0.33 kcal mol^–1^, while the Gibbs free-energy difference was approximately 0.16 kcal mol^–1^, with the all-anti structure being marginally lower in free energy. These small differences indicate that the calculations do not support a pronounced energetic preference for either of the two representative ligand-shell arrangements.

Table 3 presents the total distances between surface gold atoms (1–12) for optimized geometries within six *n*-*m* segments. The distance between central and surface atoms is approximately half the length of the *n*-*m* segment. It turns out that the Au_12_Pt core of Au_24_Pt^0^-Bu is significantly more elongated in one direction than the Au_13_ core of Au_25_^0^-Bu, consistent with the stronger Jahn–Teller distortion previously reported for Au_24_Pt clusters.^17,21^ Both clusters are elongated in a similar way; however, the differences in distances between central and core atoms are significantly larger for Au_12_Pt (Figure S8). The observed differences arise from the distinct electronic structures of Au_13_ and Au_12_Pt cores.^9^ Both optimized structures deviate from the perfect *I*_*h*_ geometry, confirming the non-octet rule model.^17,21^ Their cores are elongated along one of the six directions (defined by the positions of the six pairs of surface atoms), while the remaining directions are contracted. Both optimized structures deviate from ideal *I*_*h*_ symmetry and exhibit characteristic symmetry-lowering distortions associated with non-octet superatomic configurations.

In the representative optimized structures examined here, the stronger Jahn–Teller distortion of the Au_12_Pt core is accompanied by larger deviations of some ligand chains from an ideal *all-trans* arrangement. This observation is qualitatively consistent with the FT-IR results, which indicate a greater contribution of *gauche*-like conformational environments in the Pt-containing Bu systems. However, the calculations were not designed to determine thermodynamic conformational populations or to establish a causal relationship between the core distortion and a particular ligand conformation. In the *all*-*trans* conformation, hydrocarbon ligand chains interact with each other through van der Waals interactions. Therefore, the structure is better described as interacting regions of hydrocarbon chains separated by empty regions rather than as a uniformly distributed, directionless alkanethiolate layer. The stronger deformation of the Au_12_Pt core may influence the spatial arrangement of the attached ligands and thereby modify the intermolecular interactions within the ligand shell. Such structural differences may contribute to the increased contribution of *gauche*-like conformations inferred from the FT-IR measurements. Accordingly, the optimized structures are used here as qualitative models for interpreting the experimental trends rather than as predictions of the preferred ligand-shell conformation in solution.

### Qualitative Interpretation of the Core Distortion

The following discussion should be regarded as a qualitative interpretation intended to rationalize the DFT-derived core geometries rather than as a rigorous electronic structure treatment.

The deformation of the Au_12_M (M = Au or Pt) cores can be discussed qualitatively using a crystal-field-type picture.^40,41^ The key prerequisite for such a treatment is the presence of partially filled, doubly degenerate superatomic P manifold in systems that do not satisfy the octet rule so that a certain degree of core symmetry is preserved. In conventional octahedral complexes, the Jahn–Teller effect often manifests as a tetragonal elongation, in which one of the three Cartesian axes is lengthened while the other two are shortened. This distortion lowers the symmetry from *O*_*h*_ to *D*_*4h*_ and splits some orbitals, including triply degenerate *n*p atomic orbitals of the central atom (Figure 6). In simple crystal-field terms, the splitting is rationalized by changes in the repulsion between the electrons on the atomic orbitals of the central metal and the electrons on the ligands at the vertices of the octahedron; changing the metal-ligand distances shifts the energies of orbitals oriented along or between the bond segments.^40,41^

**Figure 6:**
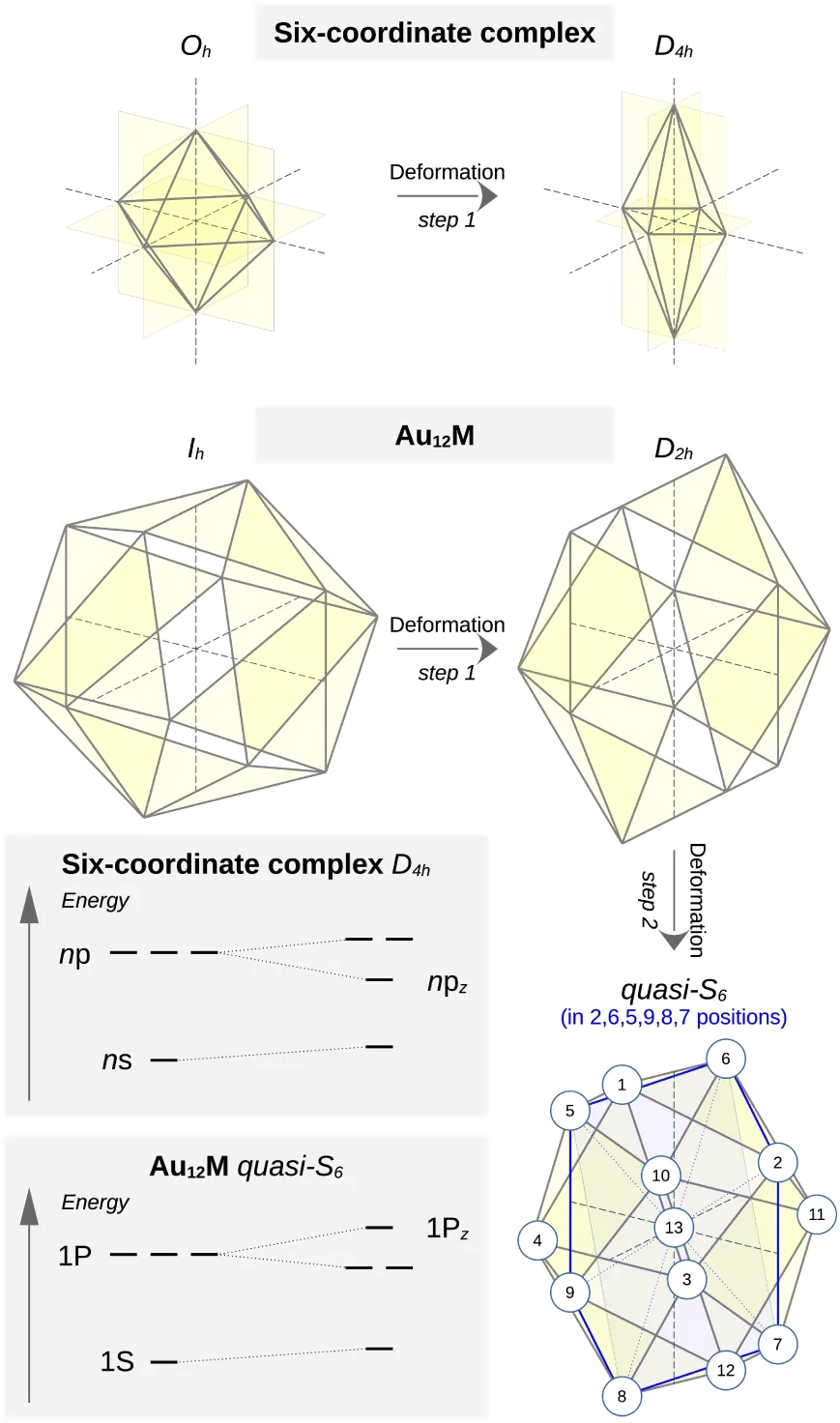
Distribution of the atomic *n*s and *n*p orbitals of central metal in tetragonally elongated octahedral complex *D_4h_* with negatively charged ligands and superatomic 1S and 1P orbitals of central metal M (gold or platinum) in *quasi*-S*_6_* deformed six-atom ring structure within the core made of central metal (M = Au or Pt) with positively charged sites (Au^+^), based on crystal-field-type description.

A similar line of reasoning can be adapted to a central atom embedded in an icosahedral core, surrounded by formally positively charged Au^+^ sites (Figure 6). In this simplified model, changes in the energies of superatomic P orbitals are attributed to changes in the electrostatic attraction between the electrons of these orbitals and the effective positive charges on the surrounding Au^+^ centers, which can be approximated by an average charge of +1/2 on each surface atom. The reasons for Jahn–Teller splitting of the 1P manifold can then be analogously viewed as arising from a distortion analogous to a tetragonal elongation: within a given plane defined by four surface atoms, two opposite directions are effectively elongated while the others are slightly compressed within *D*_*2h*_ symmetry. However, according to the group theory, double degeneracy of 1P_*x*_ and 1P_*y*_ orbitals cannot be legitimate in the *D*_*2h*_ point group. Moreover, according to the obtained DFT results, both Au_13_ and Au_12_Pt cores (from Au_25_^0^ and Au_24_Pt^0^) exhibit three segments of the same length (Table 3), whereby the postulated *D*_*2h*_ is the inaccurate model. Inspection of the optimized structures reveals that six surface gold atoms from the *n*-*m* segments, with atoms positioned at very similar distances from the central atom M (2, 6, 5, 9, 8, 7 or 11, 6, 5, 4, 8, 7 depending on the handedness), create *quasi-S*_*6*_, chair cyclohexane-like motif. To achieve this, the 2–9 segment is shortened relative to 1–12, keeping 1–12 as the longest one, while slightly shortening the remaining segments that do not participate in forming the *quasi-S*_*6*_ structure. One possible interpretation is that in non-octet superatomic configurations delocalized 1S and 1P electrons may be preferentially localized within this kind of structure to a greater extent than within the whole icosahedral core, which may provide a qualitative explanation for the apparent near-degeneracy of the 1P manifold.

### Possible Implications for Chemical Reactivity

Within the qualitative framework outlined above, the stronger Jahn–Teller distortion of the Au_12_Pt core may influence the spatial distribution of the frontier superatomic orbitals relative to Au_25_^0^. In particular, the doubly degenerate HOMO of Au_24_Pt^0^ is expected to be more sensitive to symmetry-lowering effects than the corresponding electronic states in Au_25_^0^. Together with previously reported differences in the electronic structure of Au_24_Pt and Au_25_,^9,20,21^ this suggests that platinum incorporation modifies not only the geometry of the cluster core but also its electronic response. The structural changes identified by DFT calculations may be accompanied by changes in ligand-shell organization. FT-IR measurements indicate a greater contribution of *gauche*-like conformations in Au_24_Pt^0^-Bu relative to Au_25_^0^-Bu, suggesting that platinum incorporation affects both the metal core and the surrounding ligand environment. Such combined electronic and structural effects may influence the accessibility of the cluster surface and consequently its chemical reactivity. Although the present study does not directly probe nucleophilicity or substrate binding, the proposed interpretation is qualitatively consistent with literature reports describing enhanced activity of Au_24_Pt clusters in reactions involving H_2_O, H_2_O_2_, and proton-coupled processes.^1,42^ In contrast, Au_25_ clusters have frequently been reported to exhibit distinct reactivity patterns toward less polar substrates such as CO_2_ and O_2_.^15,23^ These observations suggest that platinum incorporation modifies the interaction of atomically precise gold clusters with their chemical environment.

Such differences in reactivity may also contribute to the well-documented difficulties associated with the oxidative purification of Au_24_Pt/Au_25_ mixtures. In particular, a higher susceptibility of Au_24_Pt to oxidizing agents could promote partial decomposition during peroxide treatment, leading to the formation of secondary products and, thereby, complicating the isolation of pure Au_24_Pt samples.

## Conclusions

Several distinctive features associated with neutral Au_24_Pt^0^ clusters have been identified through a combined experimental and computational investigation of Au_24_Pt^0^/Au_25_^0^ mixtures. By integrating ICP-MS, UV-Vis, FT-IR, electrochemical measurements, and DFT calculations, reproducible differences between the platinum-containing mixtures and the corresponding Au_25_ reference systems could be identified and related to differences in electronic structure and ligand-shell organization. From a practical perspective, these results also indicate that Pt-related spectroscopic and electrochemical effects can remain experimentally observable in cluster mixtures without the complete isolation of the Pt-containing component.

The results demonstrate that platinum incorporation modifies both the electronic and structural properties of atomically precise gold clusters. Au_24_Pt^0^ exhibits distinct spectroscopic and electrochemical signatures, while DFT calculations reveal a more pronounced Jahn–Teller distortion of the Au_12_Pt core compared to the Au_13_ core of Au_25_^0^. These differences are accompanied by FT-IR changes consistent with differences in ligand-shell organization, although the calculations do not establish a unique preferred ligand conformation.

The combined experimental and theoretical results support a picture in which platinum incorporation influences not only the superatomic electronic structure but also the molecular architecture of the entire cluster. The proposed crystal-field-type interpretation provides a qualitative framework for understanding how symmetry lowering within the metal core may propagate to the ligand shell and ultimately affect the cluster reactivity.

Taken together, these findings highlight that the consequences of single-atom platinum doping extend far beyond a simple modification of metal composition. Instead, platinum incorporation induces interconnected electronic, structural, and conformational changes that collectively shape the behavior of atomically precise gold clusters and provide new insight into structure-property relationships in heterometallic superatomic systems.

## Experimental Section

### Materials

Gold­(III) chloride trihydrate HAuCl_4_·3H_2_O, chloroplatinic acid hydrate H_2_PtCl_6_·*x*H_2_O, butane-1-thiol (BuSH), hexane-1-thiol (HxSH), *n*-tetraoctylammonium bromide (TOABr), and NaBH_4_ were purchased from *Sigma-Aldrich* (Darmstadt, Germany). All other chemicals and solvents were purchased from *Avantor* (Gliwice, Poland). All reagents and solvents were of analytical grade. Argon was obtained from *Air Products* (Warsaw, Poland). Water was distilled and passed through a Milli-Q purification system (*Merck Millipore*). Glassy carbon electrodes were purchased from *BasInc* (West Lafayette, USA). Platinum counter electrode and Ag/AgCl electrode were purchased from *Redoxme AB* (Norrköping, Sweden).

### Details of the Syntheses

HAuCl_4_ (about 200 mg, a maximum of 250 mg) and TOABr (about 350 mg) were dissolved in 15 mL of tetrahydrofuran (THF) in the 250 mL round-bottom flask. For Au_24_Pt-containing products, H_2_PtCl_6_ was added additionally. In the case of using the HAuCl_4_:H_2_PtCl_6_ mixture of precursors, the necessary mass of H_2_PtCl_6_ (about 40 mg) was calculated using the HAuCl_4_ weighed mass and molar ratio Au:Pt (Ξ) ≈ 4:1. The red solution was stirred for approximately 60 min. Then about 350 μL of BuSH or HxSH was added at once. The necessary volume of thiol was calculated using the HAuCl_4_ weighed mass and molar ratio thiol:Au ≈ 6:1. The mixture turned colorless within approximately 1 h, then the bubbling solution of NaBH_4_ (about 200 mg) in 5 mL of cold distilled water was added at once. The mixture warmed up for a while and darkened due to the sudden formation of clusters and nanoparticles. The necessary mass of NaBH_4_ was calculated using the HAuCl_4_ weighed mass and molar ratio Au:NaBH_4_ ≈ 1:10 for pure Au_25_ and Au:NaBH_4_ ≈ 1:12 for Au_24_Pt-containing products. The mixture was left for 24 h.

### Details of the Purifications

THF was evaporated, leaving the orange oily phase on the surface of the aqueous phase. The mixture was transferred to a separatory funnel with toluene, washed with distilled water (total 500 mL) to remove polar impurities, and dried over anhydrous sodium sulfate and filtered. Au_25_^–^ (and Au_24_Pt^0^) species are formed in this step. In order to oxidize Au_25_^–^ to Au_25_^0^, the solution was concentrated and loaded onto the top of a gravity glass column packed with 60 Å silica gel. Elution was carried out with toluene, and the eluate was split into 20 mL fractions. Fractions with satisfactory UV-Vis spectra were combined, and the toluene was evaporated. The dry residue was centrifuged in Eppendorf tubes in methanol to remove excess thiols and TOABr released upon oxidation. Au_25_-Bu and Au_25_-Hx products were dissolved in toluene, concentrated, filtered, and dried under a stream of argon. The crude products containing Au_24_Pt-Bu and Au_24_Pt-Hx were sonicated (40°C) in an acetonitrile-acetone mixture (1:1) until dark orange solutions were obtained (first portions only). The solvent was then evaporated, and the dry residues were dissolved in toluene. The final products were obtained following the same procedure as for Au_25_-Bu and Au_25_-Hx. The final UV-Vis spectra (Au_25_^0^ forms and Au_24_Pt^0^-containing products) were recorded for all four vials.

### Au_24_Pt^0^-Hx Purification with H_2_O_2_

The Au_24_Pt^0^-Hx-containing product (a different sample from the one characterized in this paper) was dissolved in 40 mL of dichloromethane and combined with 5 mL of H_2_O_2_ (30% aqueous solution). UV-Vis spectra of portions of the mixture were recorded every 10 min over a period of 50 min.

### Description of MS

A Nexera X3 system coupled with an LCMS-9030 quadrupole time-of-flight mass spectrometer (*Shimadzu Corporation*) was used for FIA-MS. The mobile phase was 0.1% formic acid in acetonitrile. The pump was operated at 0.4 mL/min. Samples were dissolved in mobile phase solution. The injection volume of the samples was adjusted in the range of 0.1–1 μL for optimal signal intensity. The ESI positive mode was used with an interface voltage of +4.0 kV. The temperatures of the interface, desolvation line, and heat block were set at 300, 250, and 400 °C, respectively. The flow rates of the nebulizer gas and drying gas were set at 3 and 10 L/min, respectively. Mass spectrometry data were acquired in the range of 1000–10000 m/z with 250 ms event time. Mass spectra were recorded for Au_25_-Bu and Au_24_Pt-Bu.

### Description of ICP-MS

The Au_24_Pt-containing products (1.0 mg of the Au_24_Pt-Bu-containing sample and 0.5 mg of the Au_24_Pt-Hx-containing sample) were dissolved in 0.5 mL of 65% aqueous HNO_3_ and sonicated for 40 min at 60 °C. Subsequently, 1.5 mL of 37% aqueous HCl was added. The resulting solutions were analyzed using a *PerkinElmer* NexION 300D quadrupole mass spectrometer. Measurements were performed in triplicate for each product. The concentrations of gold and platinum in each product were determined based on a calibration curve constructed using standard solutions of the respective metals at concentrations of 5, 10, 50, and 100 μg/L.

### Description of DLS

DLS measurements were performed for four products dissolved in toluene and diluted to approximately 0.005 mg/mL. The measurements were carried out using a *Malvern Panalytical* Zetasizer Nano ZS instrument in standard quartz cuvettes with a 1 cm optical path length.

### Description of TEM

Four products were dissolved in dichloromethane to obtain visibly colored solutions. Drops (2 μL) of these solutions were deposited onto copper-coated TEM grids. After solvent evaporation, TEM images were recorded using a *Zeiss* Libra 120 TEM equipped with a thermionic LaB_6_ cathode, operating in bright-field mode at an accelerating voltage of 120 kV.

### Description of UV-Vis Spectroscopy

UV-Vis spectra were recorded using a *Thermo Fisher* Evolution 60 spectrophotometer in 1 cm quartz cuvettes. Samples were prepared by dissolving weighed amounts to obtain solutions with an approximate concentration of 0.25 mg/mL. Clusters concentrations during UV-Vis measurements for crude products (during purification) were not controlled.

### Description of FT-IR Spectroscopy

FT-IR spectra were recorded for solutions of four products prepared analogously to those used for UV-Vis measurements. Measurements were performed on a *Thermo Scientific* Nicolet iS5 FT-IR spectrometer by using air as the background.

### Description of CV and SWV Electrochemistry

For SWV and CV measurements, the products were dissolved in appropriate amounts of dichloromethane together with the supporting electrolyte NBu_4_PF_6_ to obtain solutions with approximate mass concentrations of 0.5 mg/mL for the products and 0.1 M for the supporting electrolyte. The solution concentrations during measurements could increase due to the high volatility of dichloromethane. A three-electrode setup was used, consisting of a glassy carbon working electrode, an Ag/AgCl reference electrode, and a platinum counter electrode. Prior to each measurement, the solutions were deaerated with argon for approximately 2 min. Measurements for all four products were performed using a *CH Instruments* Electrochemical Workstation CHI660C potentiostat. CV curves were recorded at scan rates ranging from 20 to 320 mV·s^–1^. SWV curves were recorded at a sampling frequency of 25 Hz.

### TD-DFT Results

The TD-DFT calculations for UV-Vis spectra simulations were performed for 40 electronic states for Au_25_^0^-Bu and 35 electronic states for Au_24_Pt^0^-Bu. The highest computed excited states had energies of 3.01 eV (Au_25_-Bu) and 3.26 eV (Au_24_Pt-Bu), corresponding to wavelengths of 412 and 380 nm, respectively.

### DFT Results

The file dft_coords.rtf was used as the input for all of the DFT calculations. All calculations were carried out using the *Gaussian* 16, revision C.01 software package using the PLGrid computational infrastructure. Visualizations of molecular orbitals and optimized molecular geometries were rendered by using *Jmol*. Details of the computational strategy are provided in the Results and Discussion section.

## Supplementary Material





## Notes

Input file for DFT calculations (PDF)
Materials; details of the syntheses and purifications; descriptions of mass spectrometry, DLS, and TEM; UV-Vis and FT-IR spectroscopy; CV and SWV electrochemistry; and TD-DFT and DFT results (PDF)

## Abbreviations

RSH: Alkanethiol
BuSH: Butane-1-thiol
HxSH: Hexane-1-thiol
Au_25_: Au_25_(RS)_18_
Au_24_Pt: Au_24_Pt­(RS)_18_
Au_25_-Bu: Au_25_(C_4_H_9_S)_18_ (Au_24_Pt-Bu analogously)
Au_25_-Hx: Au_25_(C_6_H_13_S)_18_ (Au_24_Pt-Hx analogously)
THF: Tetrahydrofuran
TOABr: *n*-Tetraoctylammonium Bromide
DLS: Dynamic Light Scattering
TEM: Transmission Electron Microscopy
UV-Vis: Ultraviolet–Visible Spectroscopy
FT-IR: Fourier Transform Infrared Spectroscopy
NIR: Near-Infrared Spectroscopy
NMR: Nuclear Magnetic Resonance
EXAFS: Extended X-ray Absorption Fine Structure
ICP-MS: Inductively Coupled Plasma *Mass* Spectrometry
CV: Cyclic Voltammetry
SWV: Square-Wave Voltammetry
DFT: Density Functional Theory
TD-DFT: Time-Dependent Density-Functional Theory
HOMO: Highest Occupied Molecular Orbital
SOMO: Singly-Occupied Molecular Orbital
LUMO: Lowest Unoccupied Molecular Orbital
