# Supplementary material for: Beyond the Metal Core: Platinum Incorporation Reshapes Redox Levels and Ligand Shells in Au 25 Thiolate Clusters
Source: ACS Omega. 2026 Sep 17;11(38):58024–34. doi: 10.1021/acsomega.6c07603 (PMC13628623; doi:10.1021/acsomega.6c07603)
Supplement: Supplementary file 1 [file ao6c07603_si_001.pdf]

Au25-Bu
Charge = 0, Multiplicity = 2, xyz coordinates in Angstroms

Au	0.052835	0.015964	-0.026523
Au	2.458788	-1.720023	0.517775
Au	1.671642	-0.785261	-2.370473
Au	-1.533993	-0.488342	-2.530883
Au	-2.583347	-1.109428	0.501729
Au	-0.053749	2.015758	-2.224317
Au	0.416632	2.667782	1.082627
Au	-1.765338	4.746537	-0.977140
Au	-2.972610	2.555599	-3.666751
Au	3.222816	2.101450	-3.476818
Au	4.894120	-0.801172	-1.857950
Au	-3.302682	-3.250362	-2.250109
Au	-0.352981	-2.784137	-4.484782
S	-0.602644	4.893065	1.100127
S	-2.955055	4.867234	-3.048398
S	-3.032138	0.263679	-4.352080
S	1.426392	3.667391	-3.258441
S	5.018411	0.555856	-3.822559
S	4.841306	-2.235564	0.058519
S	-4.179824	-2.874094	-0.067012
S	-2.537534	-3.759100	-4.463898
S	1.889791	-1.940917	-4.542743
C	-1.945095	4.564688	2.347545
C	-4.710870	-0.231667	-3.717367
H	-4.746883	-1.328575	-3.786885
H	-4.761089	0.052522	-2.661049
C	-5.839604	0.407604	-4.537041
H	-5.712023	0.124606	-5.593702
H	-5.743170	1.504899	-4.485916
C	-7.244712	-0.012610	-4.050204
H	-7.977744	0.258999	-4.826584
H	-7.285209	-1.111120	-3.958946
C	-7.660754	0.636787	-2.718124
H	-8.672765	0.321357	-2.423035
H	-6.974561	0.380132	-1.901832
H	-7.658782	1.734099	-2.805300
C	1.888418	4.665499	-1.762165
H	1.026006	5.317372	-1.569164
H	1.979375	3.979696	-0.912290
C	3.171033	5.475493	-1.947432
H	3.092575	6.122271	-2.836701
C	6.527118	1.623674	-3.526791
H	6.727380	2.113915	-4.490966
H	6.252175	2.396262	-2.799454
C	7.749655	0.829038	-3.048193
H	8.578028	1.548305	-2.924171
H	7.538949	0.412139	-2.049365
C	8.191173	-0.311928	-3.978936

H	8.387348	0.092995	-4.985770
H	7.364835	-1.032024	-4.083025
C	9.436521	-1.037069	-3.445368
H	9.754441	-1.842033	-4.122594
H	10.281275	-0.341326	-3.326187
H	9.231424	-1.488434	-2.462727
C	4.751482	-3.941886	-0.691087
H	4.335946	-4.575910	0.103731
H	4.026776	-3.906913	-1.512272
C	6.101950	-4.490155	-1.170144
H	5.927087	-5.544825	-1.449603
C	6.719011	-3.753712	-2.371163
C	-5.747220	-1.923203	-0.372588
H	-6.155194	-1.720149	0.626392
H	-5.480572	-0.959664	-0.816407
C	-6.751705	-2.675560	-1.255181
H	-6.355187	-2.712892	-2.282021
H	-7.668501	-2.062308	-1.287308
C	-7.096532	-4.103789	-0.788854
H	-7.890384	-4.499180	-1.442485
H	-6.218575	-4.750570	-0.940350
C	-7.543503	-4.184574	0.680832
H	-7.885178	-5.197557	0.936701
H	-6.713663	-3.935280	1.358371
H	-8.370007	-3.485857	0.885067
C	-2.134571	-5.583646	-4.294512
H	-1.355606	-5.785247	-5.042203
H	-1.706795	-5.737517	-3.294787
C	-3.357443	-6.483102	-4.527728
H	-3.725708	-6.344026	-5.557265
C	-4.511590	-6.282910	-3.530794
H	-4.107113	-6.281490	-2.507369
H	-4.958082	-5.288250	-3.683523
C	-5.600395	-7.359161	-3.663336
H	-6.422188	-7.182224	-2.954379
H	-6.026175	-7.367505	-4.678399
H	-5.191810	-8.361917	-3.464536
C	1.818061	-0.550304	-5.780766
H	2.760732	-0.002550	-5.643296
H	1.834983	-1.024253	-6.773272
C	0.620051	0.390621	-5.639897
H	-0.308998	-0.152876	-5.874118
H	0.532090	0.706482	-4.590401
C	0.750749	1.639120	-6.527873
H	0.874462	1.338619	-7.582133
H	1.666493	2.183752	-6.241205
C	-0.467101	2.565810	-6.382098
H	-0.364858	3.470656	-6.999118
H	-1.394780	2.050187	-6.671127
H	-0.583077	2.878672	-5.334995

Au	-2.343669	1.669225	-0.497723
Au	-1.562063	0.724490	2.409171
Au	1.643520	0.508274	2.470162
Au	2.698301	1.100199	-0.461144
Au	0.109046	-2.033034	2.144925
Au	-0.339137	-2.639794	-1.079777
Au	1.551071	-4.877089	0.895277
Au	2.939303	-2.668574	3.549819
Au	-3.233115	-1.918080	3.526314
Au	-4.873317	0.886610	1.725022
Au	3.439398	3.298617	2.211863
Au	0.514502	2.733597	4.490902
S	0.198568	-5.020149	-1.071175
S	2.880828	-4.963128	2.878313
S	3.132767	-0.379863	4.237107
S	-1.412609	-3.465811	3.428291
S	-4.971726	-0.283424	3.814293
S	-4.801409	1.882430	-0.443645
S	4.370933	2.822453	0.065639
S	2.586637	3.933671	4.355119
S	-1.691358	1.815663	4.619418
C	1.471890	-5.081637	-2.437167
H	2.001228	-4.121771	-2.434907
H	0.890339	-5.138191	-3.369029
C	2.438917	-6.263944	-2.318018
H	3.170106	-6.169467	-3.140336
H	3.005656	-6.174192	-1.377302
C	1.767579	-7.644943	-2.383139
H	1.041683	-7.731583	-1.559239
C	2.787653	-8.791114	-2.297266
C	1.665616	-5.704963	4.083884
H	1.433394	-6.709879	3.702238
H	0.750588	-5.100240	4.054985
C	2.238820	-5.765056	5.502263
H	3.162149	-6.366590	5.508690
C	1.219073	-6.355786	6.493830
C	4.806086	0.003387	3.512779
H	4.896037	1.099590	3.518648
H	4.796253	-0.338361	2.471680
C	5.931627	-0.654685	4.321991
H	5.853167	-0.322848	5.369261
H	5.778542	-1.746790	4.320002
C	7.340617	-0.328425	3.779623
H	8.081207	-0.655369	4.527056
H	7.452698	0.764731	3.692503
C	7.660982	-0.996896	2.430946
H	8.687638	-0.765298	2.109744
H	6.979908	-0.672490	1.634475
H	7.565430	-2.090406	2.505529
C	-1.886972	-4.709818	2.133727

H	-1.025817	-5.393183	2.073545
H	-1.968692	-4.190694	1.172368
C	-3.167540	-5.471882	2.492561
C	-3.336788	-6.753559	1.648189
C	-6.549731	-1.288081	3.663873
H	-6.783386	-1.609255	4.689721
H	-6.327946	-2.179789	3.065476
C	-7.717775	-0.501765	3.050587
H	-8.597038	-1.169675	3.045191
H	-7.480976	-0.280318	1.996693
C	-8.072479	0.811251	3.768163
H	-8.360609	0.592912	4.810014
H	-7.177182	1.450286	3.815692
C	-9.197272	1.573360	3.049412
H	-9.447891	2.507303	3.572802
H	-10.113420	0.966446	2.983587
H	-8.892357	1.833473	2.024084
C	-5.238797	3.667251	-0.218231
H	-5.082736	4.141477	-1.197562
H	-4.521799	4.105523	0.483969
C	-6.681731	3.844350	0.264989
H	-7.374271	3.414261	-0.476786
H	-6.823605	3.268379	1.194459
C	5.903295	1.840560	0.431501
H	6.355515	1.643049	-0.548572
H	5.594971	0.877845	0.848456
C	6.881026	2.561458	1.369893
H	6.440730	2.589911	2.378623
H	7.785759	1.932576	1.430545
C	7.267594	3.992945	0.949710
H	8.016464	4.370776	1.663692
H	6.385104	4.644330	1.048778
C	7.817799	4.094016	-0.482530
H	8.185080	5.107874	-0.695865
H	7.035613	3.866455	-1.220638
H	8.649676	3.390275	-0.642165
C	1.915007	5.639055	3.983827
H	1.437291	5.977269	4.915279
H	1.142880	5.527308	3.214918
C	3.005982	6.617459	3.534889
H	3.831821	6.606338	4.263944
H	3.421179	6.269100	2.575183
C	2.472100	8.058340	3.376216
H	3.319569	8.709214	3.107034
H	2.103422	8.417792	4.351790
C	1.363111	8.200956	2.317541
H	1.092605	9.256571	2.170393
H	0.450852	7.658062	2.601204
H	1.692029	7.793600	1.350963
C	-1.576156	0.431831	5.850242

H	-2.523640	-0.116821	5.747082
H	-1.560037	0.908365	6.841772
C	-0.384600	-0.509799	5.677164
H	0.552426	0.046056	5.838930
H	-0.353055	-0.867513	4.638098
C	-0.468363	-1.709142	6.635890
H	-0.531241	-1.345661	7.675647
H	-1.401176	-2.262157	6.434029
C	0.732061	-2.654952	6.483908
H	0.681428	-3.488109	7.198904
H	1.681090	-2.121165	6.640243
H	0.757391	-3.078459	5.470318
C	-7.029323	5.323789	0.513474
C	-8.461531	5.499822	1.044206
H	-8.698488	6.558519	1.221985
H	-9.197216	5.103356	0.328144
H	-8.594833	4.957426	1.992629
H	-6.311196	5.746739	1.235490
H	-6.900508	5.892030	-0.422624
C	3.454209	6.326677	-0.694815
C	4.849481	6.968110	-0.718296
H	2.678801	7.105257	-0.607064
H	3.360788	5.688396	0.198577
H	5.024335	7.578908	0.179792
H	5.627567	6.191738	-0.758101
H	4.974696	7.616701	-1.598779
H	2.295798	-9.773130	-2.344463
H	3.514650	-8.736477	-3.122078
H	3.350660	-8.740645	-1.352497
H	1.189230	-7.721657	-3.319768
C	7.942315	-4.486244	-2.944381
H	5.950865	-3.629339	-3.153337
H	7.011206	-2.736925	-2.067512
H	8.372244	-3.937785	-3.793937
H	8.728536	-4.594119	-2.181546
H	7.672997	-5.495536	-3.291654
C	-3.466124	-6.499307	0.136867
H	-4.317568	-5.838294	-0.073782
H	-2.569932	-6.016228	-0.277420
H	-3.618099	-7.444045	-0.405927
H	-2.478054	-7.420043	1.838939
H	-4.230824	-7.289547	2.005864
H	-3.142726	-5.743506	3.560153
H	-4.027756	-4.799065	2.347101
H	4.006703	4.780190	-2.121360
C	1.753635	-6.373438	7.935024
H	1.016660	-6.797699	8.631856
H	2.673231	-6.973663	8.006499
H	1.993604	-5.354399	8.274000
H	0.291040	-5.763263	6.447634

H	0.953487	-7.379164	6.179961
H	2.516330	-4.748057	5.820073
H	-2.999344	-7.525792	-4.460003
H	6.815656	-4.503906	-0.330289
C	-2.973634	5.694143	2.446438
C	-3.936978	5.484875	3.638304
C	-4.727198	4.163352	3.598413
H	-1.421628	4.436102	3.305169
H	-2.451277	6.657163	2.564478
H	-3.537506	5.758440	1.501802
H	-4.638831	6.334187	3.665823
H	-3.355229	5.529030	4.574158
H	-5.451710	4.117836	4.424657
H	-5.285976	4.061421	2.658958
C	-1.740083	5.556980	-4.295457
C	-1.447321	7.055844	-4.118874
C	-0.407429	7.381701	-3.034285
C	-0.103445	8.884434	-2.934499
H	-0.820982	4.962064	-4.238770
H	-2.217510	5.358914	-5.264434
H	-1.068406	7.424548	-5.088123
H	-2.384369	7.603457	-3.925427
H	-0.764831	7.018402	-2.057381
H	0.517971	6.826288	-3.256513
H	0.647666	9.087586	-2.157297
H	0.282449	9.275114	-3.888709
H	-1.011376	9.453539	-2.683159
H	-2.409444	3.606290	2.088684
H	-4.070487	3.288853	3.692691

Au24Pt-Bu

Charge = 0, Multiplicity = 1, xyz coordinates in Angstroms

Pt	-0.004148	-0.097730	0.097003
Au	2.361543	-1.662104	0.352124
Au	-0.639929	-2.963251	-0.315225
Au	0.412782	-1.018275	-2.634187
Au	0.491037	-1.834357	2.395792
Au	2.372304	0.948533	1.681418
Au	3.414282	-1.868367	3.620944
Au	3.873715	-1.517930	-2.797966
Au	1.813483	-4.646555	1.742875
Au	1.921489	1.011812	-4.641458
Au	2.251515	1.053346	-1.285174
Au	-4.078091	-3.719677	-0.301460
Au	-1.828026	-3.750051	-3.243719
Au	-2.323583	1.508521	-0.153622
Au	0.571506	2.736037	0.367295
Au	-0.400735	0.779706	2.879586
Au	-0.554979	1.622337	-2.271934
Au	-2.427219	-1.091755	-1.581271

Au	-3.517946	1.905937	-3.328747
Au	-3.922631	1.308801	2.885928
Au	-1.707913	4.612674	-1.553748
Au	-2.069439	-1.347838	4.671324
Au	-2.297248	-1.295887	1.351460
Au	3.946385	3.584659	0.240724
Au	1.758534	3.669996	3.251615
S	3.601247	-0.653391	-5.009341
S	3.215145	-4.267360	3.648731
S	4.234117	2.432885	-1.848881
S	4.013744	0.446847	3.439430
S	-3.856524	-4.762444	-2.448341
S	-0.450648	-3.079165	4.370854
S	4.429552	-2.400473	-0.645824
S	0.507381	-5.123146	-0.192736
S	0.212298	-2.877688	-4.177789
S	-3.621584	0.423957	5.076216
S	-3.123052	4.267414	-3.453910
S	-4.369147	-2.620946	1.823434
S	-4.141272	-0.400079	-3.219978
S	3.689696	4.748070	2.318193
S	0.280267	2.723072	-4.340949
S	-4.448081	2.222660	0.738794
S	-0.401990	4.968590	0.410335
S	-0.204095	2.739849	4.286406
C	5.646012	1.287828	-1.465559
C	4.316204	-4.245368	-0.864427
C	-0.856253	-6.266894	0.370453
C	4.913286	-4.617915	2.942637
C	5.565405	0.261920	2.421048
C	-5.179243	-3.885417	-3.423736
C	1.491233	-4.074425	-3.550449
C	5.103073	0.448836	-5.167414
C	-1.393770	-4.363597	3.405703
C	-5.677658	-1.361293	1.442431
C	-4.351792	4.067070	0.962798
C	1.047947	6.018323	-0.084241
C	-4.783742	4.877596	-2.854151
C	-5.629046	-0.325991	-2.089190
C	5.085212	4.066284	3.363316
C	-1.539573	3.866804	3.651192
C	-5.214622	-0.529274	5.319280
C	1.275141	4.085259	-3.546882
H	5.421449	0.749259	-0.537601
C	6.989763	2.015991	-1.390213
H	5.653037	0.548278	-2.278251
H	3.288862	-4.505443	-1.136053
H	4.509987	-4.672980	0.128265
C	5.320576	-4.767947	-1.898313
C	-1.466201	-7.001852	-0.826486

H	-1.610357	-5.661978	0.884314
C	6.023606	-4.357675	3.969029
H	5.042235	-3.962859	2.072204
H	4.913043	-5.662466	2.599782
C	6.706738	-0.364317	3.232634
H	5.809405	1.273920	2.074537
H	5.327093	-0.353680	1.543738
H	-5.155715	-2.829237	-3.143793
H	-6.134129	-4.319835	-3.093023
C	-4.991187	-4.024166	-4.936512
C	1.155622	-5.531100	-3.879487
H	1.579067	-3.925501	-2.469558
H	2.436068	-3.764297	-4.020360
H	5.030976	1.229260	-4.398935
H	5.007596	0.934029	-6.149607
C	6.423795	-0.319543	-5.058829
H	-1.635450	-3.934679	2.424627
C	-2.663118	-4.865309	4.096736
H	-5.276106	-0.669872	0.694938
H	-5.811666	-0.793264	2.373359
H	-3.615781	4.283763	1.741543
C	-5.722076	4.661723	1.302257
C	0.634713	7.414269	-0.555081
H	1.617937	5.489919	-0.858484
H	1.675613	6.072524	0.816226
C	-4.775230	6.364865	-2.491569
H	-5.090691	4.263194	-1.997753
H	-5.481873	4.674906	-3.679431
C	-6.936838	0.011991	-2.815988
H	-5.415762	0.406526	-1.301235
H	-5.684047	-1.318375	-1.621527
H	5.119917	4.715080	4.250771
C	6.436023	4.048851	2.637868
H	4.798464	3.057031	3.680528
C	-1.268670	5.337386	3.982683
H	-2.469750	3.513655	4.120352
H	-1.614623	3.709884	2.569662
H	-5.215846	-1.367036	4.610749
C	-6.473287	0.335951	5.149261
H	-5.144878	-0.942480	6.334806
H	1.588840	3.748874	-2.549594
H	0.559336	4.909402	-3.415120
C	-7.008768	-1.954446	0.978528
H	-0.421043	-6.966233	1.097183
H	-0.676377	-5.182045	3.243067
H	-3.965312	4.473577	0.017739
C	2.487319	4.507476	-4.384417
C	-8.070458	-0.849822	0.812166
H	-6.856405	-2.480500	0.021468
H	-7.359244	-2.711904	1.698094

C	-6.085040	-3.258993	-5.705586
H	-4.004021	-3.617898	-5.209920
H	-4.993696	-5.087742	-5.225579
C	-2.774917	-7.717070	-0.443566
H	-1.670527	-6.282417	-1.633247
H	-0.736765	-7.723872	-1.228375
C	2.248980	-6.486576	-3.373257
H	0.199045	-5.793424	-3.400481
H	1.015717	-5.649437	-4.966268
C	5.512717	-6.293420	-1.789637
H	4.966391	-4.494281	-2.905503
H	6.294476	-4.269107	-1.764284
C	7.411496	-4.308609	3.302358
H	5.833058	-3.394869	4.469886
H	6.000760	-5.129475	4.754272
C	8.030779	-0.452236	2.445609
H	6.863160	0.213841	4.158129
H	6.392238	-1.373144	3.539168
H	7.175659	3.613832	3.332029
H	6.367958	3.358886	1.781149
C	6.937072	5.410317	2.133704
C	1.864708	8.326105	-0.728999
H	0.084501	7.323284	-1.506230
H	-0.062702	7.865496	0.169765
C	-2.398732	6.252210	3.481560
H	-1.136467	5.457778	5.070182
H	-0.322313	5.640679	3.507368
C	-5.648480	6.191940	1.472184
H	-6.437136	4.411955	0.500554
H	-6.107666	4.199353	2.225824
H	-7.336458	-0.302612	5.407326
C	-6.513361	1.622226	5.997565
H	-6.577374	0.605385	4.085804
C	3.184572	5.762891	-3.817291
H	3.195623	3.665740	-4.406781
H	2.173338	4.695259	-5.424245
H	-7.759390	-0.178056	-2.105302
C	-7.036225	1.464899	-3.310302
H	-7.086966	-0.683925	-3.657701
C	-6.183597	6.861802	-2.114039
H	-4.080338	6.525926	-1.650495
H	-4.385610	6.953417	-3.337787
C	8.164401	1.023672	-1.317256
H	7.109365	2.663551	-2.274483
H	6.995309	2.682860	-0.512498
C	-3.387015	-5.916613	3.233487
H	-2.416831	-5.287036	5.084604
H	-3.335576	-4.010308	4.267446
C	7.638187	0.623787	-5.152356
H	6.452675	-0.861488	-4.098888

H	6.477166	-1.083115	-5.851635
H	-7.469741	2.132688	5.800542
C	-6.357670	1.374866	7.507026
H	-5.719340	2.302870	5.650832
C	-2.139112	7.733723	3.796772
H	-2.497076	6.125140	2.392408
H	-3.354313	5.933333	3.931653
C	-9.370362	-1.370911	0.179672
H	-8.283567	-0.402785	1.797607
H	-7.651376	-0.039160	0.193914
C	8.974185	-0.131075	-5.058206
H	7.594188	1.186164	-6.099680
H	7.574647	1.371306	-4.344559
H	4.168460	5.863728	-4.302581
C	2.380433	7.057661	-4.033503
H	3.387281	5.611241	-2.743156
C	9.526067	1.732159	-1.256546
H	8.037678	0.373891	-0.438405
H	8.128150	0.362086	-2.197278
H	8.721742	-1.109729	2.996786
H	7.842697	-0.947521	1.477770
C	8.704069	0.912731	2.219508
C	1.509853	9.671182	-1.381130
H	2.328159	8.493121	0.257229
H	2.617637	7.804587	-1.340121
C	8.296217	5.290700	1.424821
H	6.194688	5.831717	1.437879
H	7.009476	6.110995	2.982154
C	-3.364348	-8.513264	-1.618442
H	-2.592440	-8.386181	0.414447
H	-3.501419	-6.959588	-0.106276
C	-5.900326	-3.355798	-7.228740
H	-7.077068	-3.649641	-5.421811
H	-6.058565	-2.201376	-5.395472
C	1.930650	-7.958770	-3.676277
H	3.218847	-6.211153	-3.819596
H	2.347432	-6.350211	-2.285371
C	6.362830	-6.855861	-2.939817
H	5.987698	-6.524722	-0.821366
H	4.530936	-6.789920	-1.779495
C	8.529088	-3.983348	4.306305
H	7.619055	-5.270379	2.804194
H	7.397465	-3.541802	2.509501
C	-4.729559	-6.349272	3.841915
H	-3.567280	-5.495537	2.230841
H	-2.732487	-6.793229	3.092869
C	-8.436186	1.808850	-3.843082
H	-6.768907	2.141943	-2.480946
H	-6.287553	1.635971	-4.099103
C	-6.193021	8.338906	-1.689466

H	-6.861325	6.716042	-2.971475
H	-6.579671	6.237738	-1.296790
C	-7.031283	6.827842	1.683613
H	-4.993756	6.431463	2.323022
H	-5.169386	6.629896	0.582536
H	-7.204993	8.671370	-1.418256
H	-5.540977	8.501782	-0.818424
H	-5.829309	8.983365	-2.503600
H	-6.955491	7.917681	1.805042
H	-7.691353	6.630281	0.825725
H	-7.518861	6.418772	2.581310
H	-2.956019	8.370776	3.427179
H	-2.043467	7.898158	4.880554
H	-1.204956	8.071831	3.322929
H	6.516369	-7.939325	-2.834356
H	5.872850	-6.676533	-3.908892
H	7.351893	-6.374089	-2.971745
H	9.829115	0.558757	-5.100480
H	9.042229	-0.694694	-4.115338
H	9.077065	-0.851285	-5.883584
H	-6.684477	-2.801310	-7.763980
H	-4.926765	-2.940518	-7.529242
H	-5.936207	-4.403278	-7.564504
H	-8.486348	2.847913	-4.199914
H	-8.708597	1.151604	-4.682833
H	-9.199539	1.682424	-3.059753
H	9.653712	0.805558	1.677220
H	8.067962	1.590347	1.634652
H	8.914265	1.404380	3.181577
H	9.511720	-3.943899	3.814648
H	8.348843	-3.007838	4.783100
H	8.578393	-4.740084	5.103391
H	8.654930	6.268147	1.072554
H	9.059543	4.871527	2.098447
H	8.221419	4.624519	0.551490
H	-4.302494	-9.011198	-1.333510
H	-3.576885	-7.843766	-2.464278
H	-2.660986	-9.287062	-1.962740
H	-10.118145	-0.571273	0.077696
H	-9.178558	-1.784109	-0.822533
H	-9.811829	-2.172730	0.790417
H	-5.226604	-7.106897	3.218508
H	-4.591611	-6.773992	4.847988
H	-5.405028	-5.485016	3.929316
H	2.937967	7.939696	-3.688501
H	2.153475	7.198448	-5.101232
H	1.425437	7.040428	-3.489878
H	2.393462	10.319178	-1.471729
H	1.098449	9.516952	-2.389836
H	0.754504	10.209477	-0.788669

H	2.720422	-8.624951	-3.299193
H	0.982028	-8.255345	-3.203303
H	1.831847	-8.128288	-4.759057
H	-6.480884	2.308077	8.074600
H	-7.108692	0.656246	7.870392
H	-5.361740	0.973970	7.743489
H	10.352735	1.009983	-1.192952
H	9.684960	2.350442	-2.153063
H	9.585343	2.392423	-0.378839
