# Supplementary material for: Beyond the Metal Core: Platinum Incorporation Reshapes Redox Levels and Ligand Shells in Au 25 Thiolate Clusters
Source: ACS Omega. 2026 Sep 17;11(38):58024–34. doi: 10.1021/acsomega.6c07603 (PMC13628623; doi:10.1021/acsomega.6c07603)
Supplement: Supplementary file 2 [file ao6c07603_si_002.pdf]

Beyond the Metal Core: Platinum Incorporation Reshapes Redox Levels and Ligand Shells in Au_{25} Thiolate Clusters

Konstanty Zdunek, Tatiana Korona, Agnieszka Więckowska*
Faculty of Chemistry, University of Warsaw,
Pasteura 1 02-093 Warsaw, Poland

*awiec@chem.uw.edu.pl

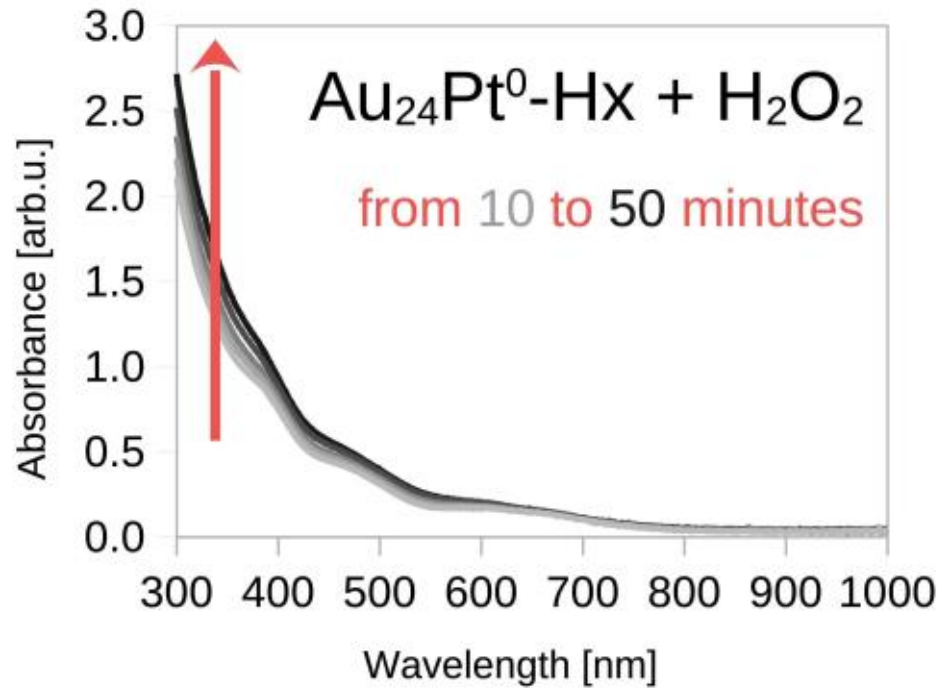

Figure S1. UV-Vis spectra (in dichloromethane) of $Au_{24}Pt^{0}$-Hx-containing product exposed to H_2O_2, recorded at 10-minute intervals over 50 minutes after mixing the reagents.

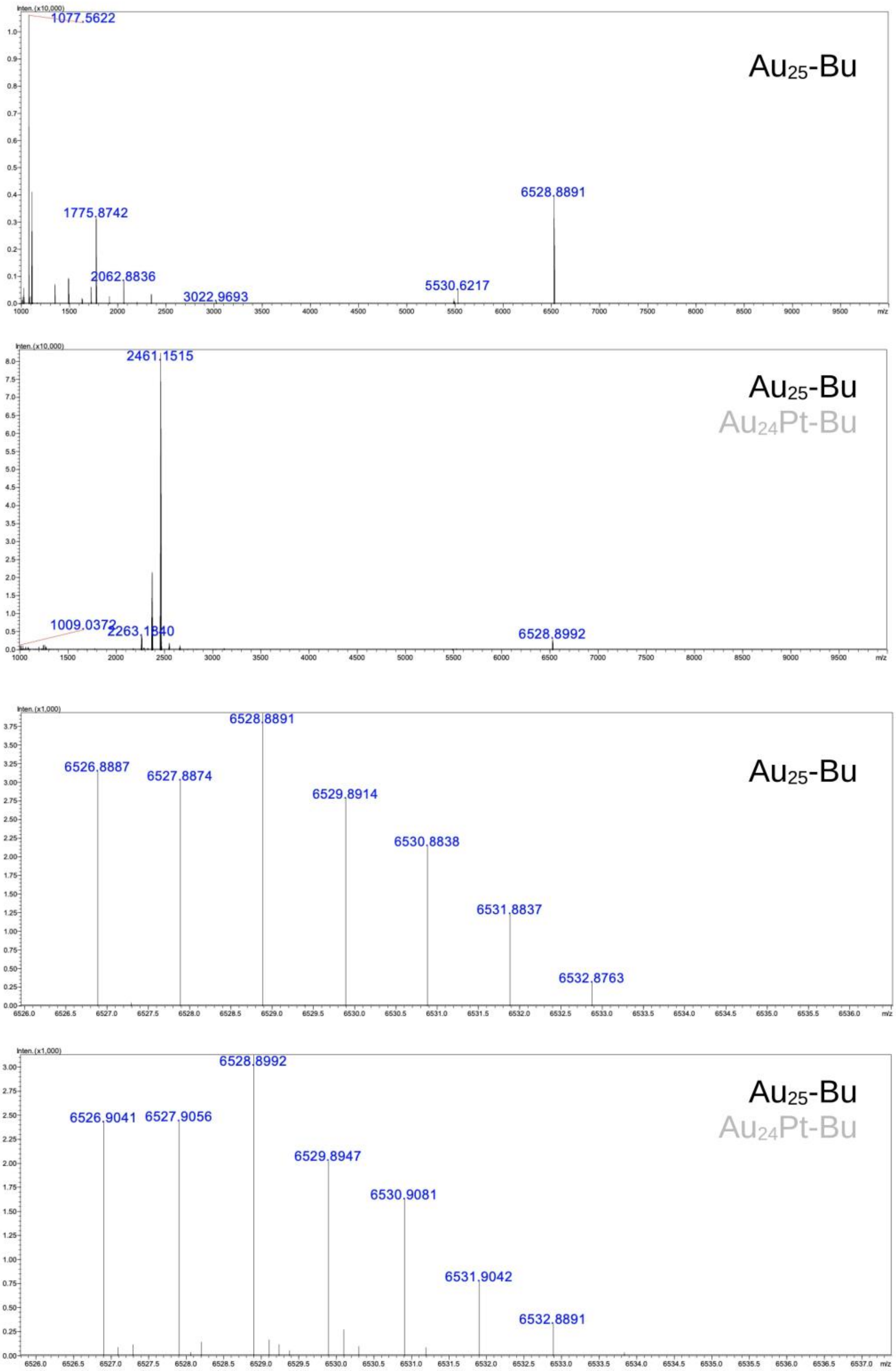

Figure S2. ESI-QTOF-MS analysis of the Au_{25}-Bu reference sample and the BuSH-protected Pt-containing sample. In the Pt-containing sample, the observed molecular-ion envelope is consistent with Au_{25}-Bu, whereas no unambiguous molecular-ion signal attributable to $Au_{24}Pt$-Bu was detected under the applied ESI-MS conditions.

The experimentally determined Ξ values for the platinum-containing products are related to the molar ratios $\frac{n_{Au_{25}}}{n_{Au_{24}Pt}}$ and $\frac{n_{Pt_k}}{n_{Au}}$ (Equation S1). To estimate $\frac{n_{Pt_k}}{n_{Au}}$ values (Equation S2) Γ

parameter (the function of the efficiency of gold conversion for Au_{25} syntheses) is unnecessary if the component $\frac{a}{c}$ (proportional on the amount of assumed Au_k atoms) is excluded in the final equation (Equation S3).

$$\frac{n_{Au_{25}}}{n_{Au_{24}Pt}} = \frac{1}{25}\frac{1}{\frac{1}{\Xi}-\frac{n_{Pt_k}}{n_{Au}}} - \frac{24}{25} \qquad \text{Equation S1}$$

$$\underbrace{\left(\frac{1}{\Gamma}-1\right)\cdot\frac{1}{25}\cdot M_{Au_{25}}}_{a}\cdot n_{Au} + \underbrace{\left(\frac{1}{\Gamma}-1\right)\cdot\left(M_{Au_{24}Pt}-\frac{24}{25}\cdot M_{Au_{25}}\right)}_{b}\cdot n_{Pt} + \qquad \text{Equation S2}$$
$$-\underbrace{\left[\left(\frac{1}{\Gamma}-1\right)\cdot\left(M_{Au_{24}Pt}-\frac{24}{25}\cdot M_{Au_{25}}\right)+M_{Pt}\right]}_{c}\cdot n_{Pt_k} = 0$$

$$\frac{n_{Pt_k}}{n_{Au}} = \frac{a}{c}+\frac{b}{c}\frac{1}{\Xi} \approx \frac{b}{c}\frac{1}{\Xi} \qquad \text{Equation S3}$$

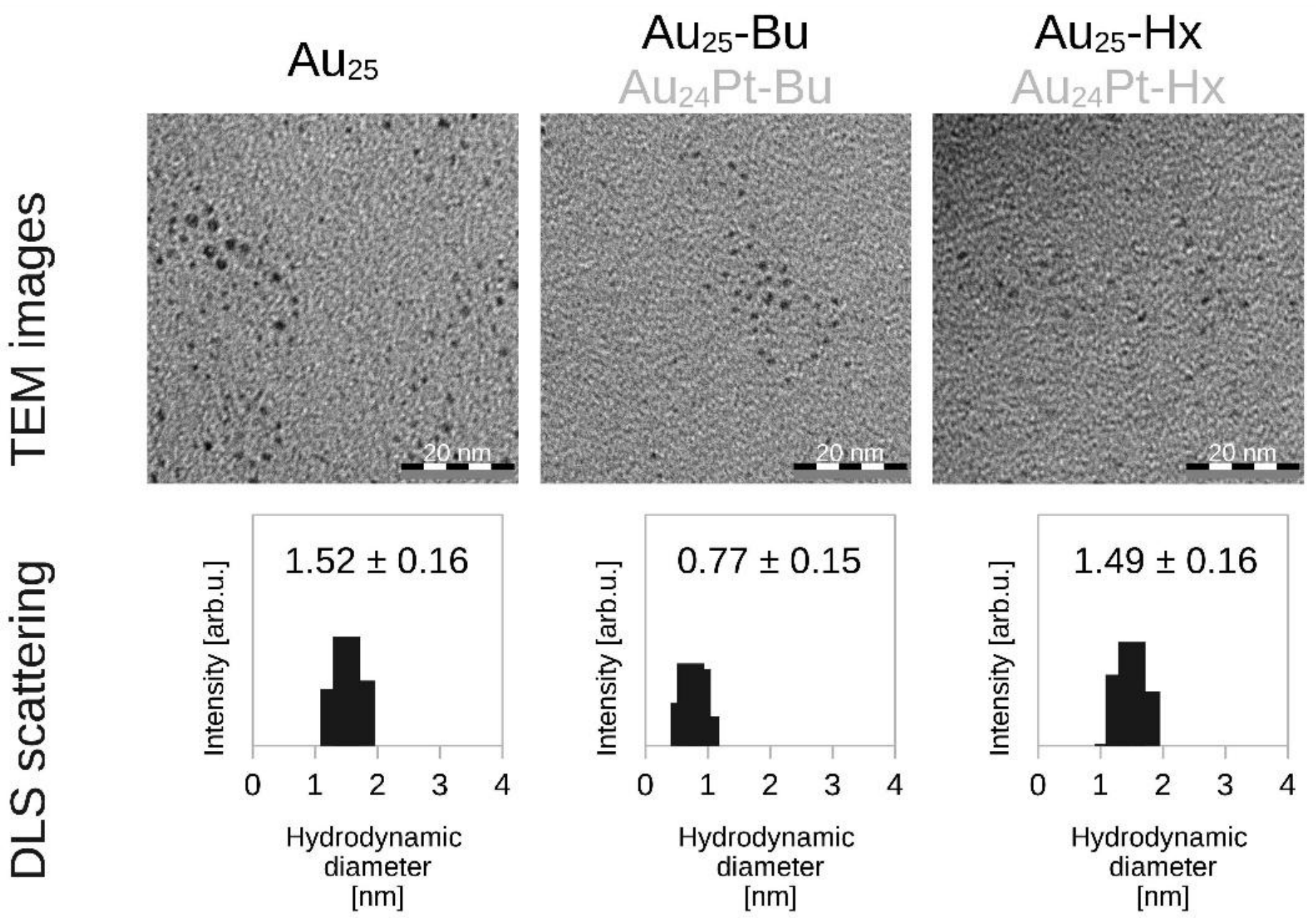

Figure S3. TEM images (bright-field imaging in solid phase) and DLS results (0.005 mg/ml toluene solutions, 1 cm optical cell) with average hydrodynamic diameters for Au_{25} samples (averaged, TEM for Au_{25}-Bu), $Au_{24}Pt$-Bu and $Au_{24}Pt$-Hx-containing samples.

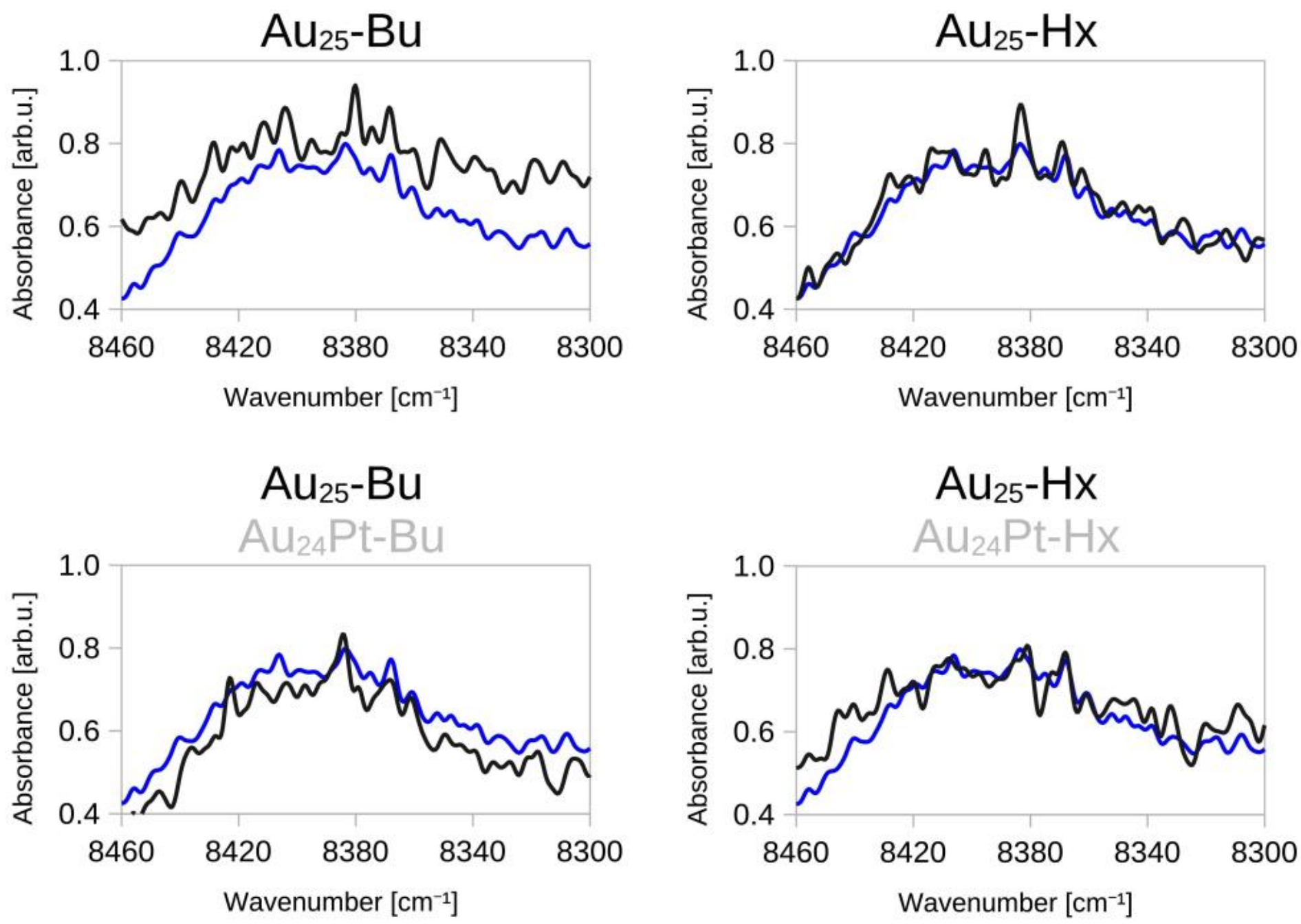

Figure S4. FT-IR spectra in selected NIR region of Au_{25}-Bu, Au_{25}-Hx, and Au_{24}Pt-Bu and Au_{24}Pt-Hx (both in mixture), black curves (0.25 mg/mL in hexane), compared to hexane, blue curves.

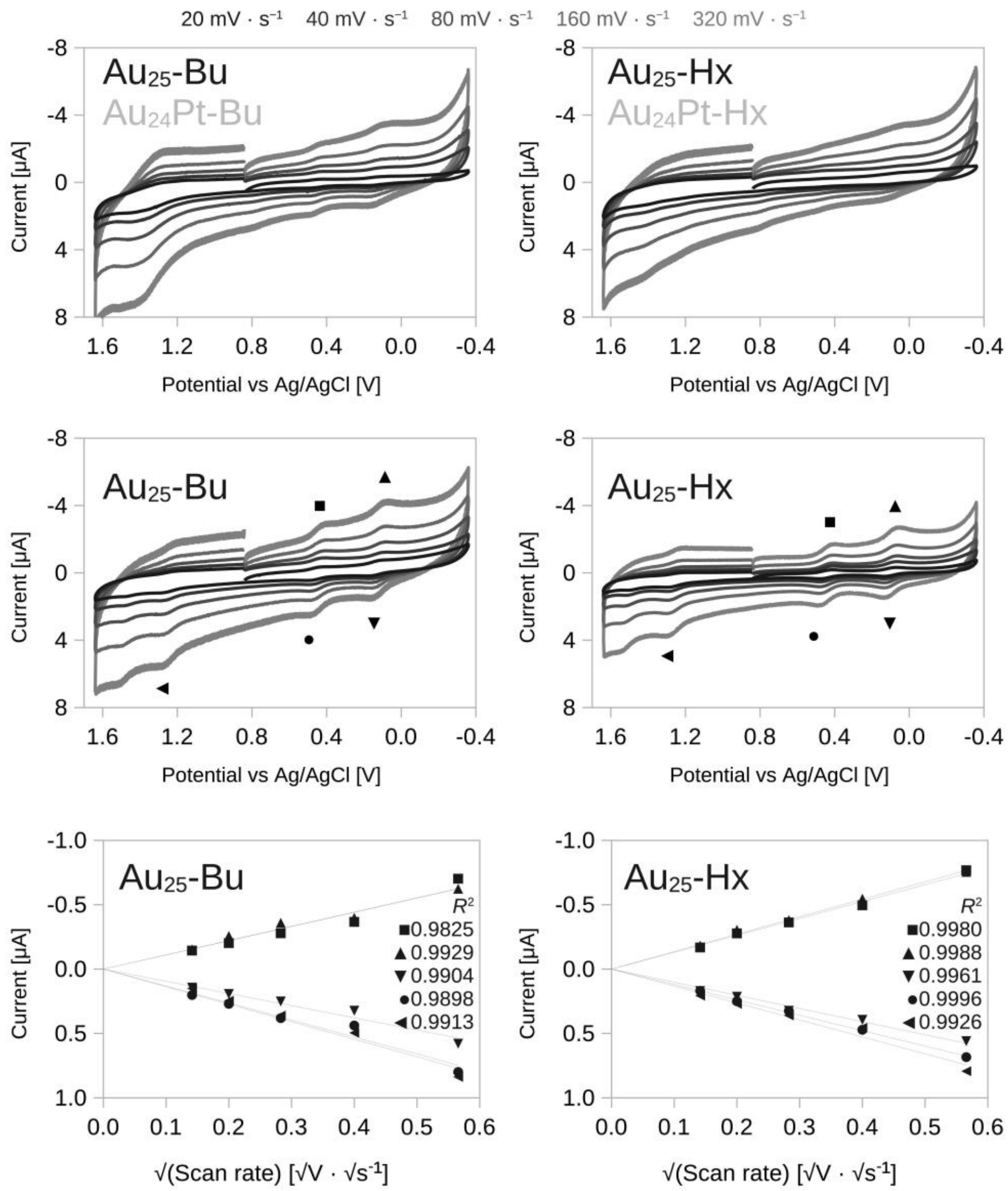

Figure S5. CV voltammograms for $Au_{24}Pt$-Bu, $Au_{24}Pt$-Hx-containing products and Au_{25}-Bu and Au_{25}-Hx clusters (0.5 mg/ml + 0.1 M NBu_4PF_6 dichloromethane solutions, from 20 mV · s^{-1} to 320 mV · s^{-1}, started in cathodic direction); linear fits of selected Au_{25} signals to the Randles–Ševčík equation, including R^2 values.

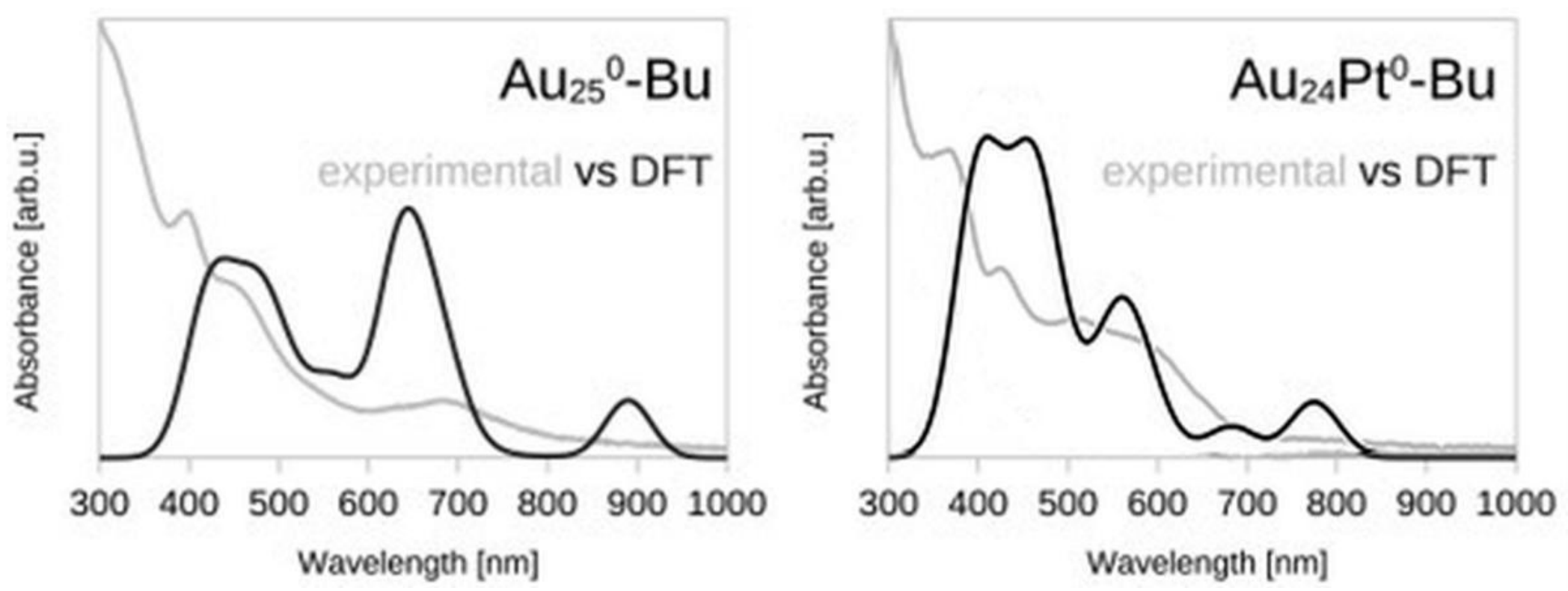

Figure S6. Qualitative comparison of the simulated TD-DFT UV-Vis spectra of Au_{25}^{o}-Bu and $Au_{24}Pt^{o}$-Bu with the corresponding experimental Bu-protected spectra. In the $Au_{24}Pt$ panel, the experimental curve corresponds to the estimated $Au_{24}Pt$-Bu spectral contribution obtained by subtraction of the appropriately scaled Au_{25}-Bu spectrum from the $Au_{24}Pt$-Bu-containing mixture.

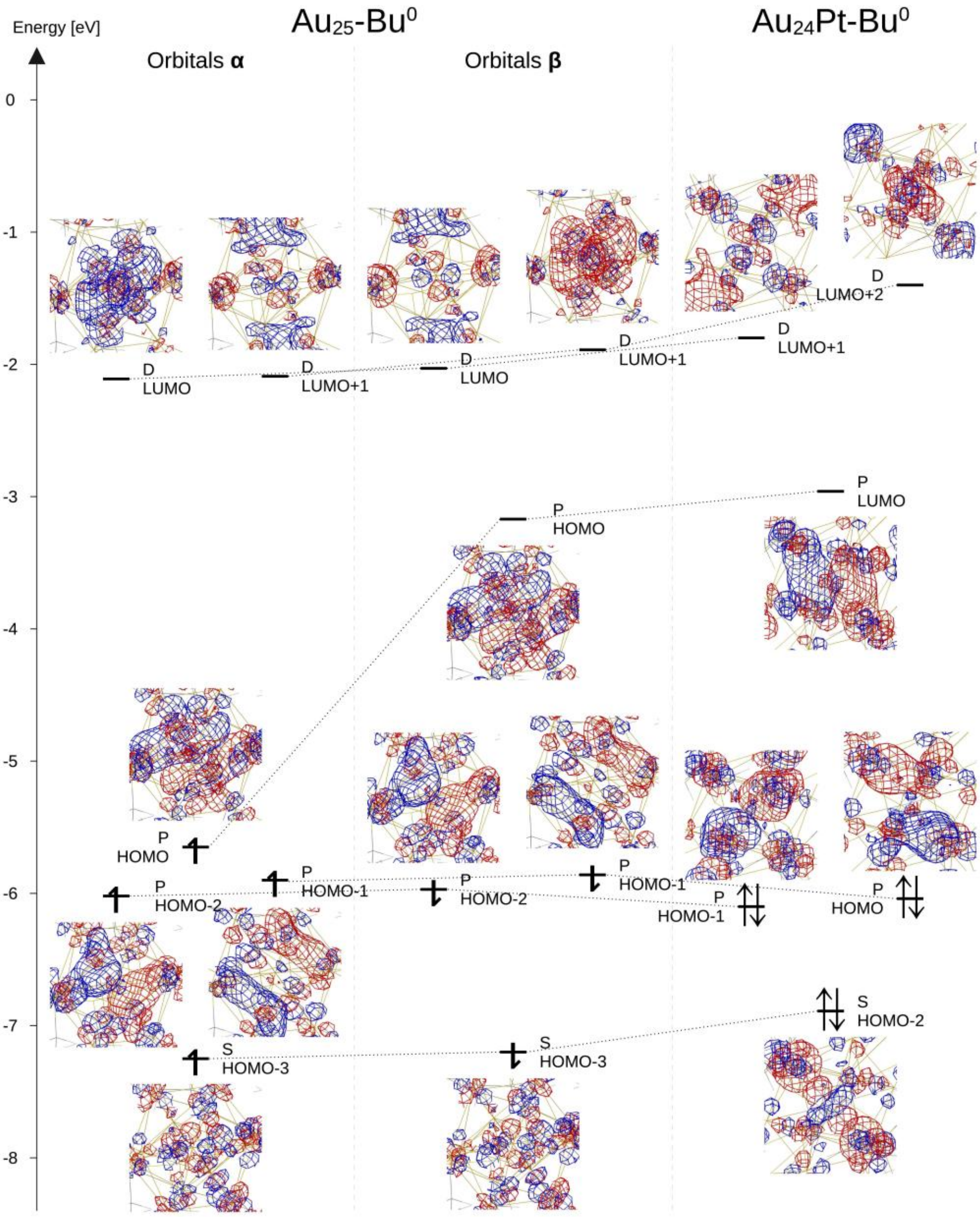

Figure S7. Energy-level diagram of the S, P, and D orbitals of Au_{25}^{0}-Bu and Au_{24}^{0}Pt-Bu clusters with DFT-optimized geometries; superatomic orbital contours within the Au_{13} and Au_{12}Pt cores are highlighted. The assignments are based on the spatial character of the calculated orbitals and do not imply a fixed energetic position of the corresponding superatomic states relative to the HOMO.

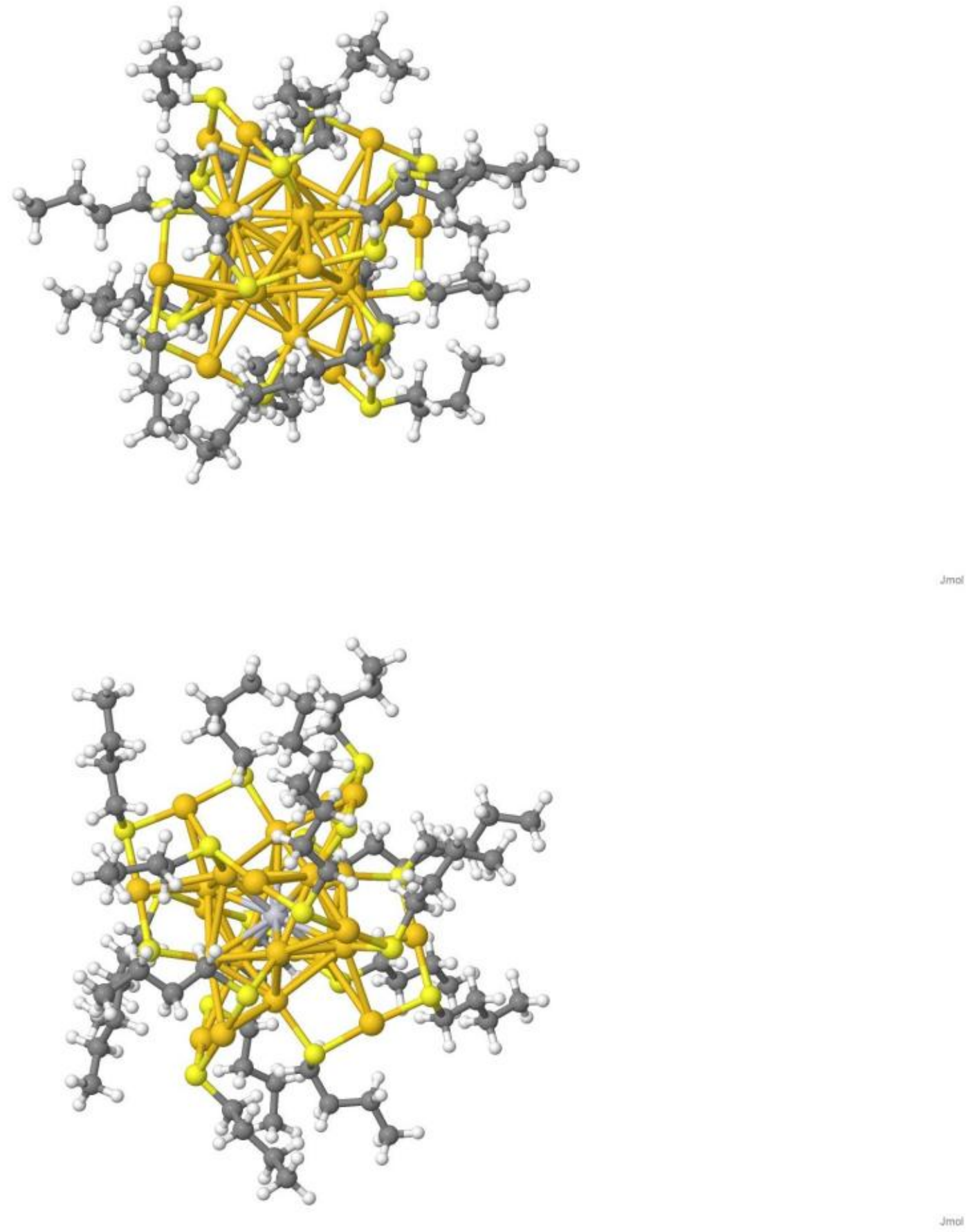

Figure S8. Au_{25}^{0}-Bu and $Au_{24}Pt^{0}$-Bu models with DFT-optimized geometries in vacuum; colors: Au – orange, Pt – grey, S – yellow, C – black, H – white.

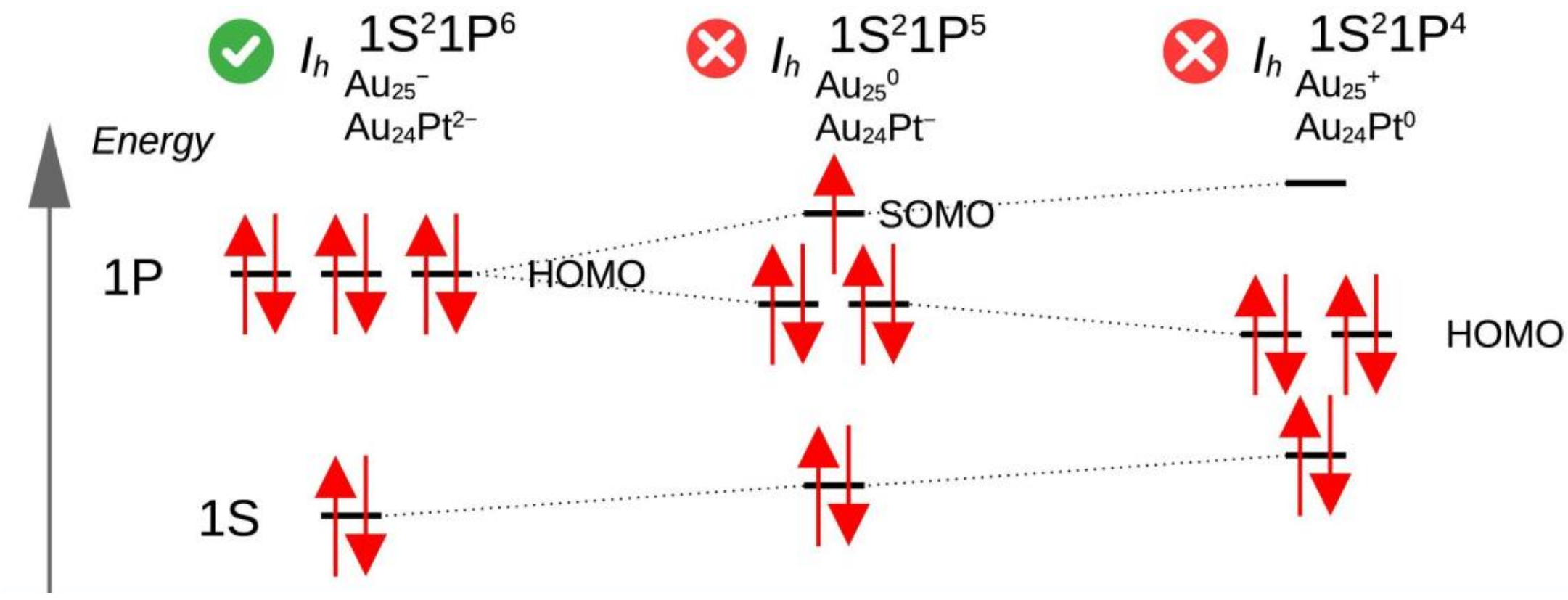

Figure S9. Distribution of delocalized electrons on superatomic orbitals in Au_{25} and $Au_{24}Pt$ clusters, illustrating the deformation of the clusters (loss of *Ih* symmetry) in $1S^21P^5$ and $1S^21P^4$ configurations.

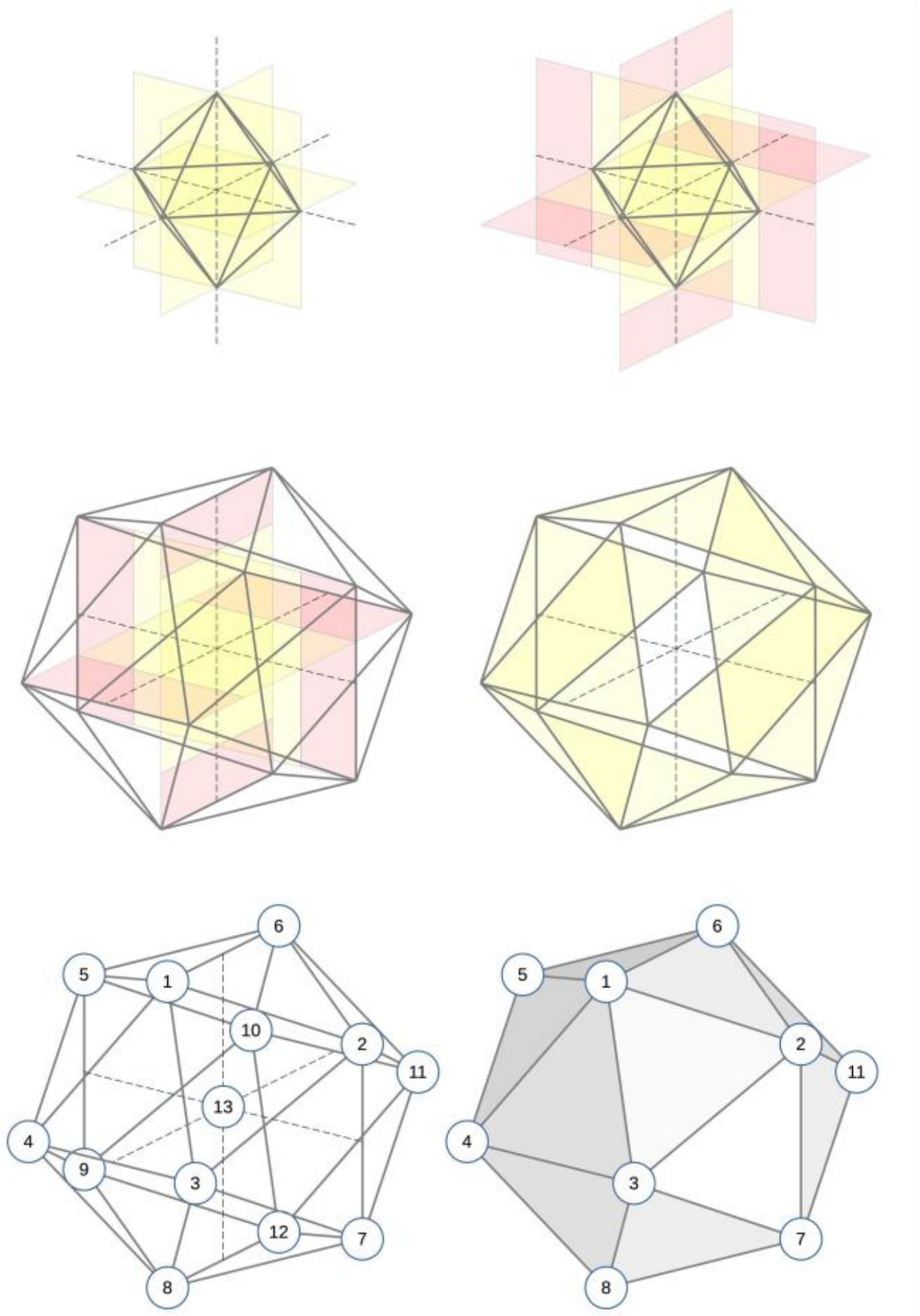

Figure S10. Evolution from perfect octahedral *Oh* (at the top) to the perfect icosahedral *Ih* shape (at the bottom).
